# Epigenetic and Transcriptional Reprogramming in 3D Culture Models in Breast Cancer

**DOI:** 10.3390/cancers17233830

**Published:** 2025-11-29

**Authors:** Laura Cecilia Flores-García, Karla Rubio, Eloisa Ibarra-Sierra, Macrina B. Silva-Cázares, Carlos Palma-Flores, César López-Camarillo

**Affiliations:** 1 Posgrado en Ciencias Genómicas, Universidad Autónoma de la Ciudad de México, San Lorenzo 290. Col. del Valle, Ciudad de Mexico C.P. 03104, Mexico; lcfloresg@hotmail.com (L.C.F.-G.); cpalma@secihti.mx (C.P.-F.); 2 Laboratorio Internacional EPIGEN, Consejo de Ciencia y Tecnología del Estado de Puebla (CONCYTEP), Instituto de Ciencias, Ecocampus, Benemérita Universidad Autónoma de Puebla, Puebla C.P. 72570, Mexico; epigen.concytep@puebla.gob.mx; 3 Departamento de Investigación, Instituto Estatal de Cancerología Dr. Arturo Beltrán Ortega, Acapulco C.P. 39610, Guerrero, Mexico; comite.etica@cancerologiagro.gob.mx; 4 Unidad Académica Multidisciplinaria Región Altiplano, Universidad Autónoma de San Luis Potosí, Matehuala C.P. 78700, Mexico; macrina.silva@uaslp.mx

**Keywords:** breast cancer, three-dimensional culture, 3D, Epigenetics, miRNAs, LncRNAs, histone code

## Abstract

Breast cancer remains the leading cause of cancer-related death in women worldwide. This disease is characterized by its molecular and phenotypic heterogeneity, which hinders the development of effective therapies. While two-dimensional (2D) monolayer cell cultures are widely used, they are insufficient to reproduce the characteristics of the tumor microenvironment, thus limiting our understanding of cancer biology. In this context, three-dimensional (3D) models have emerged as representative tools that more accurately reproduce tissue architecture, cell signaling, and nutrients and oxygen gradients. These cellular models offer greater similarity to primary tissues, improving the study of relevant biological processes. Although 3D cultures provide numerous advantages in cancer research, there is no unified model that standardizes the matrix type and parameters such as gelation time or porosity, hindering the reproducibility and interpretability of the data. This review integrates evidence from various studies to evaluate the effect of epigenetic variations generated by 3D culture methods, which are regulated by mechanotransduction and, consequently, by signaling pathways such as integrin/FAK-ILK/Rho-YAP derived from interactions of cells with extracellular matrix-enriched scaffolds. This affects processes such as DNA methylation, histone coding, and the regulation of non-coding RNAs such as microRNAs (miRNAs), long non-coding RNAs (lncRNAs), and circular RNAs (circRNAs) in different molecular subtypes of breast cancer. Overall, the evidence highlights that 3D culture methods are not equivalent but rather generate distinct epigenetic signatures at the non-coding RNA level that influence the proliferation, differentiation, therapeutic resistance, and metastatic potential of tumor cells. Furthermore, the evidence suggests that histone coding patterns, primarily through the reduction of acetylation marks, are conserved regardless of the type of 3D culture. In summary, the study highlights that the microarchitectural and compositional characteristics of 3D scaffolds are key determinants of epigenetic plasticity.

## Simple Summary

This review was conducted to address the complexities of breast cancer research, which is often limited by two-dimensional cell cultures that fail to accurately represent the tumor microenvironment. Although three-dimensional (3D) models are generally superior, the absence of standardized methodologies has resulted in inconsistent findings. The objective of this review was to systematically analyze how different 3D culture methods influence the epigenetic reprogramming of tumor cells, focusing on changes in DNA methylation, histone modifications, and chromatin organization, as well as the differential expression of various types of non-coding RNA. Our findings demonstrate that different 3D culture methods are not equivalent and yield distinct epigenetic signatures. This underscores the urgent need to standardize protocols and provide detailed reports on the properties of culture media to enhance reproducibility, which is crucial for identifying new therapeutic targets.

## 1. Introduction

According to GLOBOCAN data (2022), breast cancer is one of the most significant diseases worldwide due to its high incidence, with 2,296,840 cases, while its mortality rate makes it the leading cause of cancer-related death in women, accounting for 666,103 cases [1]. However, despite advances in treatment, such progress has been insufficient to improve patient survival due to the heterogeneity of the disease, which has been classified into four molecular subtypes based on the expression of membrane receptors: luminal A (Estrogen Receptor positive [ER+]), luminal B (ER+ and Progesterone Receptor positive [PR+]), Human Epidermal Growth Factor Receptor 2 HER2 (HER-2+), and triple-negative (TNBC) (ER-, PR-, and HER-2-) [2,3]. Therefore, it is essential to implement diagnostic models that can help develop more effective therapeutic strategies and enhance their translational application across the different subtypes of the disease.

In this context, most knowledge about cancer biology has been generated from two-dimensional (2D) monolayer cell cultures. However, it must be recognized that this model is insufficient to replicate fundamental characteristics of the tumor microenvironment, such as cell–cell and cell–matrix interactions, nutrient and oxygen gradients, cell polarity, morphology, intrinsic cell plasticity, pluripotency, and epithelial–mesenchymal transition (EMT) [4,5,6,7,8,9]. These limitations directly impact epigenetic patterns and gene expression, affecting signaling processes and generating altered responses that are not entirely comparable to tumor tissue, often leading to an overestimation of the therapeutic potential of anticancer drugs.

Consequently, three-dimensional (3D) culture systems have been implemented, which allow the replication of tissue architecture and exhibit greater similarity in gene expression compared to primary tumor tissues [4,6,7,10,11,12]. Although 3D cultures offer numerous advantages in cancer research, there is no unified model for breast cancer studies due to variations in the selection of 3D culture systems, support materials, gelation times, scaffold concentrations, and porosity, which may differ according to the molecular subtype. These differences generate variability in the epigenetic and transcriptional patterns of cells, hindering reproducibility and, consequently, the identification of stable molecular patterns for drug screening [6,11,13,14,15,16].

Thus, our objective is to systematically analyze the available experimental evidence on epigenetic reprogramming reported in 3D cultures compared to traditional monolayer cultures. This analysis aims to determine whether consistent patterns exist in DNA methylation, histone modifications, and the expression of non-coding RNAs across different 3D culture methodologies. Ultimately, this approach seeks to identify conserved epigenetic biomarkers that could be extrapolated for the diagnosis and treatment of breast cancer.

## 2. Systematic Search

A systematic search was conducted in August 2025 using the PubMed database, restricting the results to publications from 2015 to the present. However, relevant studies published before 2015 were also included through manual retrieval after reviewing the reference lists of selected articles.

A total of 294 results were obtained using the following combination of search terms: *(breast cancer AND 3D culture) AND epigenetic*, *AND DNA methylation*, *AND chromatin*, *AND histone modification*, *AND non-coding RNA*, *AND miRNAs*, *AND lncRNAs*, *AND circRNAs*.

Inclusion Criteria

- Articles that meet at least two of the breast cancer and 3D culture search criteria.
- Experimental studies comparing epigenetic changes between 3D and 2D cultures.
- Experimental studies comparing epigenetic changes between different 3D culture methodologies.
- Studies assessing DNA methylation patterns, histone modifications, or the expression of miRNAs, lncRNAs, or circRNAs.
- Experimental or review articles describing 3D culture methods.
- Publications were released between 2015 and 2025.

Exclusion Criteria

- Articles fulfilling only one of the search criteria.
- Studies evaluating epigenetic changes solely in the context of invasion or migration assays comparing 2D vs. 3D cultures.
- Studies using breast cancer cell lines only in preliminary experiments, but not in epigenetic analyses.

**Figure 1:**
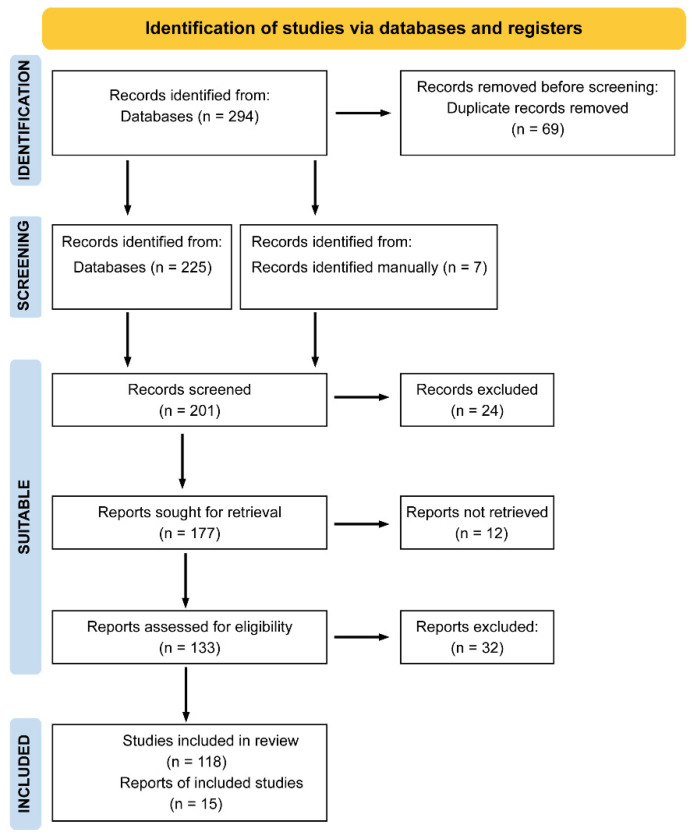
PRISMA flow diagram of study selection. A total of 294 records were identified through database searching. After removing duplicates and applying inclusion and exclusion criteria, 133 studies were finally included in the systematic review.

According to these criteria, 294 articles were initially identified. After removing 69 duplicates from overlapping search combinations, the abstracts of the remaining studies were screened. Based on this review, 24 articles were excluded for not meeting at least two search criteria. Of the remaining 177 articles, 12 could not be retrieved in full text, and 32 were excluded for meeting one or more exclusion criteria. Ultimately, 133 articles fulfilled all requirements and were included in the systematic review (Figure 1).

## 3. Types of Breast Cancer Cell Morphology in 3D Cultures

Despite the existence of different 3D culture methodologies, basic spatial organization patterns have been established. These structures lack polarity and lumen, generating three distinguishable zones: an outer layer with proliferating cells and high accessibility to oxygen and nutrients, a middle layer with quiescent cells, and a core composed of hypoxic cells undergoing apoptosis or necrosis [17,18,19,20]. This intratumoral heterogeneity generates metabolic gradients, variations in cell signaling, pH, and oxidative stress (ROS), influencing the reprogramming of epigenetic and transcriptomic patterns that directly impact the protein profile. These processes, in turn, are affected by the size of the 3D structure and the maturation of the system through successive rounds of replication and morphological changes [8,19,21,22,23,24,25,26]. It should also be noted that their 3D morphology makes it possible to distinguish non-tumor from cancer cells: the former typically form acinar structures, have a rounded shape with established polarity and lumen, and exhibit high expression of Keratin 15 (KRT15), Fatty acid-binding protein 5 (FABP5), and Anterior gradient protein 2 homolog (AGR2) [22,27].

However, in the case of 3D breast cancer cell cultures, morphological variations are observed due to the phenotypic heterogeneity derived from the molecular subtypes. This heterogeneity is represented by approximately 84 cell lines that can adopt different structural types that correlate with their gene expression signatures [2,12,23,28]. One of the earliest morphological classifications was proposed by Kenny et al. (2007) [29], which identified four main morphologies: (a) mass, characterized by a round colony outline and disorganized nuclei; (b) round, capable of forming round colonies with regularly organized nuclei; (c) grape-like, forming colonies with poor intercellular contact; and (d) stellate, with stellate projections that often connect multiple colonies [7,30,31]. The four morphologies and their associated breast cancer cell lines are summarized in Table 1.

Based on this classification, no specific morphological patterns associated with the molecular subtypes were identified. However, in the TNBC subtype, an expression profile was observed that appears to influence the formation of a 3D star-shaped structure [29]. The cellular response within 3D cultures can, nonetheless, be modulated by several scaffold-related factors, including its structure, composition, and stiffness, which have been reported to produce variable effects on tumor cell growth, migration, and other behaviors.

## 4. Types of 3D Culture Systems in Cancer

The development of 3D systems began over 30 years ago, initially based on the intrinsic capacity of cells to self-aggregate [9]. Today, the methodologies used can be divided into two major groups: the first comprises scaffold-free models, in which cells are forced to self-aggregate through direct cell–cell interactions, and the second includes scaffold-based models, which allow cells to interact with a substrate [18,26,32]. Although both systems allow cell–cell and cell–matrix interactions, they differ in terms of cellular complexity, structural organization, and functional potential [20].

### 4.1. Scaffold-Free 3D Culture Types

- Low-adherence systems:
  ○ Spheroids: These were the first 3D cultures and rely on low-adhesion plates that allow colony formation through self-assembly. They can be monocultures or co-cultures with stromal cells and can replicate hypoxia in the nucleus of the aggregate [6,33,34]. This system typically uses ultra-low adhesion (ULA) plates and poly-2 hydroxyethyl methacrylate (PolyHEMA)-coated plates [26].
  ○ Mammospheres: Aggregates formed from tissue samples, metastases, or cell lines selected for their cancer stem cell (CSC) characteristics when cultured under low-adhesion, serum-free conditions [33,35,36].
- Suspension Cultures: Based on cell–cell interactions, these are spheroids formed in flasks with gentle and continuous shaking, allowing the formation of aggregates with uniform compaction and efficient transport of nutrients and oxygen [7,20].
- Magnetic Levitation: Spheroids are formed by adding nanoparticles to a cell suspension and exposing them to a magnetic field, enabling the formation of aggregates capable of synthesizing their own extracellular matrix (ECM). These nanoparticles are composed of biocompatible materials such as iron oxide [20,26,37].
- Hanging Drop: A cell suspension is placed on the lid of a culture dish, which is then inverted, allowing surface tension and gravity to drive aggregate formation through intercellular adhesion and natural cell–cell interactions [12,20,26].
- Bioreactor: Using a simulated microgravity bioreactor in continuous rotation, cells remain in suspension and aggregate naturally. This method reduces mechanical stress and facilitates the homogeneous formation of spheroids [26,36,37].

In addition to these methodologies, the use of Plasmax^TM^ culture medium medium (Thermo Fisher Scientific, Waltham, MA USA) for the generation of spheroids exhibiting a metabolic profile like that of xenografts has recently been implemented [26]. The Gibco OncoPro™ tumor culture medium (Thermo Fisher Scientific, Waltham, MA USA) has also been used to facilitate derivation and long-term culture, demonstrating that approximately 85% of the samples analyzed (*n* = 20) formed tumoroids, including breast, colorectal, endometrial, and lung cancers [38].

Despite advances in scaffold-free 3D cell culture methods, they have a significant limitation: the formation of these structures relies on weak intercellular interactions. These interactions are modified by the ability of certain cells to produce ECM components, which restricts their applicability in breast cancer cell lines with variable E-cadherin or N-cadherin expression [26,36]. Furthermore, the absence of ECM prevents the activation of signaling pathways regulated by extracellular components and represents the avascular phase of tumor progression [39,40].

### 4.2. Scaffold-Based Models

- Organotypic Cultures: Originally emerging as homo-cellular aggregates composed of stem cells that self-assemble when introduced into a scaffold, facilitating cell attachment and organization, these cultures were later developed using other types of matrices, as well as hetero-cellular cultures that can exhibit cystic or solid phenotypes [6,7,26,27,41]. Although this is currently the most widely used system and the one in which the most cancer studies are conducted, it presents low reproducibility and high heterogeneity due to the use of diverse scaffolds, making the identification of therapeutic targets challenging.
- Patient-derived tumor organoids (PDTOs): 3D cellular structures obtained from tumor samples and incorporated into an extracellular matrix, which preserve primary tumor characteristics such as cellular and genetic heterogeneity, and histomorphology [34,42,43,44]. Furthermore, they can be employed as micro-physiological models that enable the identification of different cell populations, including tumor cells, myoepithelial cells, fibroblasts, and adipocytes, as well as the study of migration patterns [45].
- Xenograft-Derived Organoids (PDX): Aggregates generated from tumor cells expanded as xenografts and embedded in an extracellular matrix, maintaining the architecture, cellular heterogeneity, and molecular characteristics of the original tumor [46,47,48].
- Organ-on-a-Chip (OoC) Systems: Using microfluidic systems that allow continuous media perfusion, patient explants are embedded to recreate vascular flow, chemical gradients, and dynamic co-cultures [47,49]. These systems offer precise control over the microenvironment, enabling studies of metastasis and interactions between tissue types [50].
- 3D Bioprinting: A technology that applies 3D printing to generate functional biological tissues and models using bioinks containing living cells, growth factors, and biomatrices such as gelatin, hyaluronic acid, elastin, or spider silk proteins. This approach allows the fabrication of specific geometries and the production of vascularized mammary organoids [6,16,41]. Bioprinting methods, including inkjet, extrusion, and laser-assisted printing, enable the spatial and temporal control of chemical signals within 3D matrices [51].

**Figure 2:**
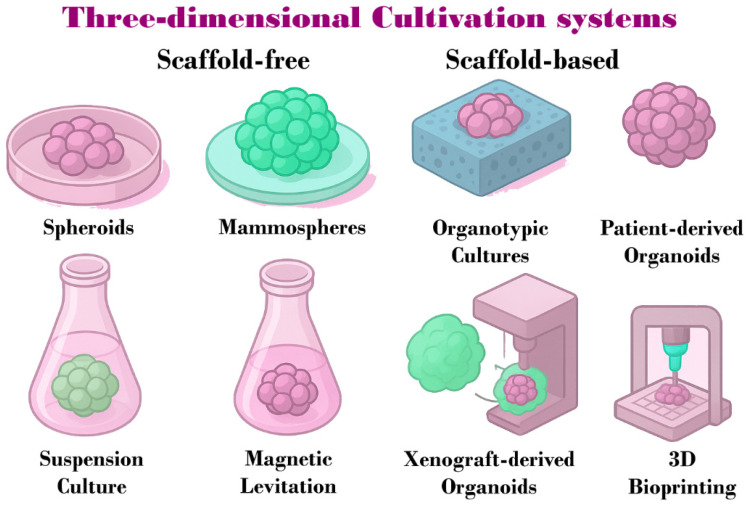
Schematic representation of 3D cultivation systems used in breast cancer research. These systems are classified into scaffold-free methods, including spheroids, mammospheres, suspension culture, and magnetic levitation, and scaffold-based methods, such as organotypic cultures, PDO, PDX, and 3D bioprinting.

Although scaffold-based 3D cultures represent a major step toward physiologically relevant models for studying cancer biology at the protein, transcriptional, and epigenetic levels, the heterogeneity of scaffold materials and the absence of standardized protocols limit reproducibility and the comparative interpretation of results, thereby hindering the identification of robust therapeutic targets. These limitations arise from the lack of standardization in scaffold composition, polymerization parameters, and physicochemical characterization. Despite the extensive literature on breast cancer 3D cultures (2145 publications according to PubMed), most studies fail to report key parameters such as batch, composition, rigidity, and porosity, which hampers systematic characterization and the establishment of common criteria. For this reason, it may be useful to explore the comparative evaluation of protocols as a research direction to promote methodological standardization and fully exploit the potential of scaffold-based models (Figure 2). Table 2 summarizes the main 3D culture methodologies and breast cancer cell lines employed in these studies.

## 5. Types of Scaffolds or Matrices Used in 3D Culture

**Figure 3:**
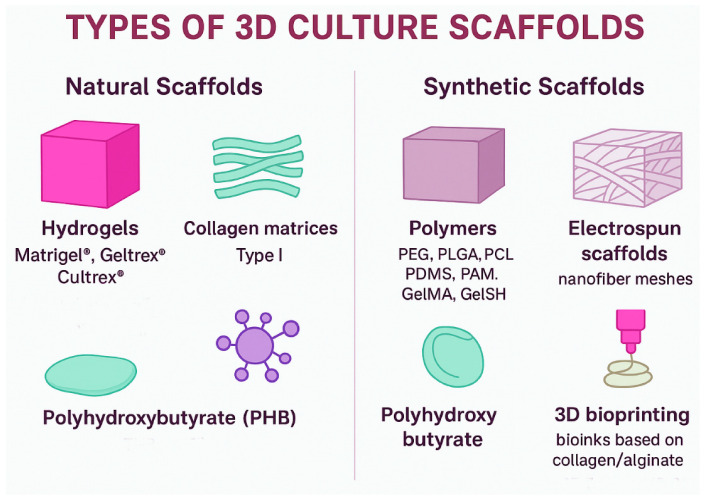
Classification of 3D culture scaffolds. Natural scaffolds include hydrogels (Matrigel^®^, Geltrex^®^, Cultrex^®^), collagen type I matrices, and PHB, which provide bioactive signals and structural support. Synthetic scaffolds comprise polymers (PEG, PLGA, PCL, PDMS, PAM, GelMA, GelSH), electrospun nanofiber meshes, polyhydroxybutyrate, and 3D bioprinting systems based on bioinks, offering tunable physical and mechanical properties for breast cancer 3D models.

The main objective of using scaffolds in 3D culture is to provide a substrate that mimics the ECM, offering both structural support and biochemical and mechanical properties comparable to those of the native tissue [18,41]. For a scaffold to be effective, it must fulfill specific physical requirements: high porosity and an interconnected structure to enable efficient cell adhesion and the exchange of nutrients and waste, as well as sufficient mechanical strength with stiffness values like those of the breast tissue EMC [78]. Based on these features, scaffolds can be classified into the following categories (Figure 3):

Natural Scaffolds: Derived from proteins and polysaccharides present in the cellular matrix. This group includes:

- Hydrogels: Solid scaffolds of natural or synthetic origin, such as those derived from Engelbreth-Holm-Swarm (EHS) tumor extracts. These scaffolds have a high laminin content and form the extracellular matrix (lrECM) gels commercially available as Matrigel^®^ (Corning Incorporated, Tewksbury, MA, USA), Geltrex^®^ (Thermo Fisher Scientific, Waltham, MA USA), and Cultrex^®^ (R&D Systems, Minneapolis, MN, USA) [18,27,79]. Among the main disadvantages of natural hydrogels, reproducibility remains a major concern. Due to their tumor-derived origin, batch-to-batch variability arises from differences in the proportions of collagen IV, laminin, entactin, and perlecan [18,27,79]. For instance, variations in collagen I and fibrinogen content alter the biomechanical strength of the scaffold, which in turn regulates the capacity for self-renewal and quiescence in MCF-7 cells. A mechanical force of approximately 45 Pa activates Integrin β1/3 receptors, triggering stem cell signaling through the cytoskeleton/AIR axis, whereas a higher force (~450 Pa) induces quiescence by arresting cell cycle signaling through receptor 2 containing the discoidin domain/signal transducer and activator of transcription 1/cyclin-dependent kinase inhibitor 1B (DDR2/STAT1/P27) [80]. Additionally, 3D breast cancer cell cultures may fail due to antigenic reactions associated with the murine origin of the matrix [18,26,27]. Beyond these intrinsic limitations of natural hydrogels, compositional variability must also be considered. For example, the commercial variant of Matrigel^®^ with reduced growth factor content (GFRM) was developed to minimize inconsistencies in growth factor composition; however, this alteration affects proliferation rates as result of changes in gene expression [81]. Similarly, combining type I collagen with Matrigel^®^ or Cultrex^®^ modifies the biomechanical properties of the scaffold [72,82].
- Collagen matrices: These scaffolds can be composed of type I or type IV collagen, each generating distinct structural networks. Type I collagen forms thick, rigid fibers that create high stiffness gradients (~1–10 kPa), making it suitable for evaluating invasion and EMT. In contrast, type IV collagen polymerizes into a branched network with lower stiffness (~100–300 Pa), facilitating the study of cell polarity and differentiation [83,84]. Moreover, polymerization conditions can be modulated by adjusting ionic strength, pH, and temperature [6]. One of the main advantages of collagen matrices is that cells can degrade collagen through the secretion of proteolytic enzymes, allowing dynamic matrix remodeling during proliferation, migration, and infiltration [85]. However, the use of these scaffolds may be influenced by cell subtype, as HER-2+ tumors have been associated with higher levels of collagen deposition [41]. Most studies employ collagen matrices as a support for heterotypic co-cultures; for example, Blyth et al. (2023) [34] used collagen to co-culture luminal cells (MCF-7 and T47D) with myoepithelial cells and stromal fibroblasts. Similarly, Singh et al. (2020) [86] co-cultured fibroblasts with TNBC spheroids, demonstrating increased Extracellular signal-regulated kinase 1/2 (ERK1/2) phosphorylation dependent on the stiffness of the collagen matrix. Therefore, the use of collagen-based scaffolds remains undercharacterized in terms of gene expression profiling in breast cancer cell cultures.
- Other Biomaterials: Natural proteins and polysaccharides, such as fibrin, alginate, hyaluronic acid, elastin, amyloid fibers, recombinant spider silk, proteoglycans, and glycosaminoglycans, are important in various applications [6,32,41]. For instance, fibrin has a short polymerization time, which facilitates a homogeneous distribution of cells and makes it less vulnerable to degradation by proteases [87]. Alginate, in contrast, can adjust its crosslinking, thereby altering the mechanical properties of the scaffold [41]. Hydrogels based on hyaluronic acid can simulate the slow and controlled release of growth factors [88]. These compounds are frequently utilized to create bioinks for 3D bioprinting or in combination with other types of matrices [41].
- Polyhydroxybutyrate (PHB): PHB is a natural polymer belonging to the polyhydroxyalkanoate family, characterized by its high flexibility, biocompatibility, and biodegradability [18]. Its flexibility arises from the presence of pores ranging from approximately 30 to 400 µm. However, this type of scaffold is seldom used in breast cancer cell culture studies.
- Decellularized extracellular matrix (dECM): This scaffold is generated by removing cellular components from tissues while preserving the structural and architectural integrity of the matrix, including the 3D fibrillar network of collagens, laminins, proteoglycans, and growth factors [6,26]. Quality criteria for dECM require a DNA content of less than 50 ng of double-stranded DNA per mg of dry weight, fragment lengths under 200 bp, and the absence of nuclear material as confirmed by DAPI staining [89]. However, the decellularization method can alter ECM composition, leading to variability that hampers reproducibility. Although approximately 200 proteins are shared among patient-derived dECM, Matrigel^®^, and xenografts, each matrix also contains unique proteins [90]. In addition, dECM may trigger immunogenicity due to residual antigenic motifs and protein fragments [41].

While most scaffold-based culture methodologies rely on collagen, combinations with extracellular matrix components such as fibronectin, laminins, hyaluronic acid, alginate, fibrin, and tenascins are increasingly used to better replicate the native ECM [6,41,91].

Synthetic scaffolds: These are constructed from synthetic polymers with simple composition and tunable crosslinking properties, such as polyethylene glycol (PEG), poly(lactide-co-glycolide) (PLG), poly(ε-caprolactone) (PCL), polyacrylamide (PAM), methacryloyl gelatin (GelMA), thiolated gelatin crosslinked with PEG-4MAL (GelSH), poly(lactic-co-glycolic acid) (PLGA), and polydimethylsiloxane (PDMS) [6,27,41]. Synthetic scaffolds enable the fabrication of microspheres, allowing precise control over the chemical and mechanophysical properties of the system. They also support the incorporation of different cell types, making it possible to evaluate specific conditions and their effects on proliferation, invasion, and metabolic pathways [92]. However, breast tissue cells require biochemical signals from extracellular matrix components, which are absent in these synthetic polymers [27,41].

Table 3 summarizes the breast cancer cell lines employed with the various scaffold types described above. For each scaffold type, the table lists the corresponding cell lines used in experimental studies, providing a clear overview of the models applied to different 3D culture systems.

Although a wide variety of scaffolds are available, it is important to recognize that the response of tumor cells can be influenced by the structure, composition, and stiffness of the materials used to mimic ECM, as scaffold-dependent variations in cell growth, migration, differentiation, and drug resistance have been reported [6,41]. For instance, pore size relative to cell size can determine the extent of intercellular interactions and proliferation, thereby influencing cell adhesion, nutrient diffusion, and motility [18,28,41]. Consequently, selecting the appropriate scaffold type and composition is essential to achieve an optimal pore size relative to the specific cell line being cultured. In addition to its biological impact, matrix porosity can also pose limitations for detection methods, leading to technical and economic challenges.

Moreover, scaffold purity is another factor that can influence cell behavior. A clear example of this variability is observed in hydrogels derived from lrECM, such as Matrigel^®^, Geltrex^®^, and Cultrex^®^. Although these products share a common origin, their physicochemical and biochemical properties differ [106,107,108], as summarized in Table 4.

## 6. Mechanotransduction as a Scaffold-Type Sensor

An important consideration in 3D cultures is mechanotransduction, which regulates physiological and pathological processes by activating signaling pathways that influence tumor progression. This is initially mediated by the scaffold, which mimics the ECM. In turn, it modulates stiffness in different regions of the cell aggregate, reflecting the structural complexity of breast tissue and making it challenging to replicate in 3D cultures [6,109,110,111]. Mechanotransduction is achieved through a “molecular clutch” mechanism, regulated by scaffold stiffness, that stabilizes the binding of actomyosin filament adapter protein–integrin contractile complexes [110]. This process, known as mechanosensitivity, depends on focal adhesions (FA) that transmit mechanical forces and signaling cues through conformational changes in cytoplasmic proteins. These changes, in turn, increase nuclear pore size and promote histone deacetylation through a yet unidentified mechanism [110].

For example, it has been reported that 3D cultures of TNBC and HER-2+ breast cancers exhibit greater rigidity due to increased collagen deposition compared to luminal A and B tumors, which appear to significantly impact Wingless-related integration site (WNT)/β-catenin signaling [6,46]. Another illustration of how mechanotransduction influences cell behavior depending on scaffold type is the study by Lingard et al. (2024) [27], which demonstrated that MCF-10A cells cultured in Matrigel^®^ show higher levels of laminin-332 and collagen IV, promoting greater differentiation compared to cells grown in stiffer hydrogel matrices.

**Figure 4:**
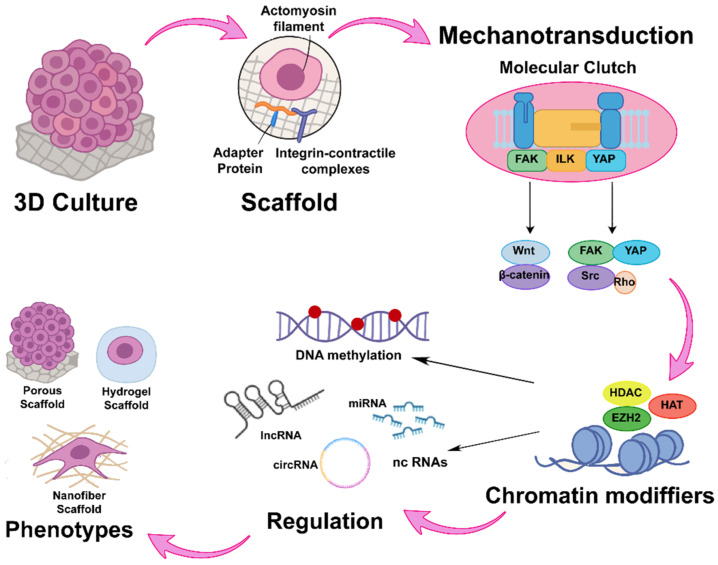
Mechanotransductive axis and its epigenetic impact in 3D models. Mechanotransduction is mediated by the “molecular clutch” complex, which consists of adaptor proteins and integrins linked to actomyosin filaments. This complex activates signaling pathways through FAK, ILK, and YAP, subsequently modulating downstream cascades such as WNT/β-catenin and SRC/Rho. These signals regulate chromatin-modifying enzymes, thereby inducing changes in DNA methylation patterns and in the expression of non-coding RNAs.

Furthermore, in other breast cancer models, rigid scaffolds have been shown to promote proliferation through collagen binding to integrin-linked kinase (ILK), which increases phosphorylation of myosin phosphatase subunit 1 (MYPT1) and suppresses signaling from Merlin, mammalian sterile 20-like kinase 1/2 (MST1/2), and large tumor suppressor kinase 1 (LATS1). This cascade, in turn, modulates focal adhesion molecules, including focal adhesion kinase 1 (FAK), the pro-oncogenic tyrosine-protein kinase SRC (SRC), and the GTPases Rac, Rho, and Ras (Figure 4) [41].

Provenzano et al. (2009) [112] also demonstrated that mammary cell phenotype is regulated by collagen matrix stiffness, with increased stiffness being directly proportional to activation of the FAK–SRC/Rho/ERK signaling axis in 3D-cultured T47D and MDA-MB-231 cells. Under low-stiffness conditions (~10 kPa), cells exhibited tubular structures, whereas under high-stiffness conditions (~44 kPa), they formed lumen-less colonies with a more protrusive phenotype, elevated FAK levels, and actin stress fiber formation due to Rho and ERK activity, which collectively induced transcriptomic changes in proliferation-associated genes.

However, in 3D cultures of T47D and MCF-7 cells embedded in PEG–heparin hydrogels, despite Rho activation, an increase in the nuclear localization of p21 has been observed with increasing matrix stiffness (2 and 20 kPa), resulting in smaller aggregate size as hydrogel rigidity increases [113]. In contrast, MCF-7 cells cultured in GelMA hydrogels exhibit an inverse relationship between Integrin β1 activity and scaffold stiffness. Under low-stiffness conditions, Integrin β1 displays higher activity, promoting activation of the transient receptor potential cation channel subfamily V member 4 (TRPV4) and cytoplasmic localization of Yes-associated protein (YAP), thereby enhancing cell proliferation [103].

Moreover, hydrogel stiffness has been shown to inversely regulate cyclin D expression and to induce variations in histone H3 acetylation. These effects are largely attributed to actomyosin contractile forces that respond to matrix stiffness, thereby modulating the expression of mechanosensitive genes. Most of these genes are regulated through laminin-associated chromatin accessibility, which in turn influences other epigenetic mechanisms such as DNA methylation and the expression of non-coding RNAs (Figure 4) [92].

Taken together, these data demonstrate that tumor cell–mediated mechanotransduction can differentially activate the molecular clutch depending on the type of scaffold employed. In addition, the intrinsic characteristics of each breast cancer molecular subtype such as integrin expression profiles and the composition of the ECM produced by the tumor cells represent crucial factors influencing scaffold-dependent responses. For instance, Luminal A tumors typically exhibit stiffness values ranging from 0.5 to 1 kPa and low expression of integrins β1 and β4, whereas Luminal B and HER-2+ subtypes display intermediate stiffness (2–5 kPa) and higher expression of integrins α6β1 and αvβ5/β4. In contrast, TNBC tumors are characterized by markedly higher stiffness (6–20 kPa) and overexpression or activation of integrins α5β1, αvβ3, and αvβ6 [114,115,116,117]. Therefore, the selection of an appropriate 3D culture methodology must consider both the specific research question and the cellular model employed, ensuring that the chosen system accurately recapitulates the biophysical and biochemical features of the tumor microenvironment under investigation. In this context, the following sections will analyze the effects of 3D culture systems and scaffold types on the epigenetic modifications experienced by tumor cells, compared with those observed in conventional 2D cultures.

## 7. Impact of 3D Culture on Tumor Cell Epigenetics

Although epigenetic changes do not alter DNA sequences, they influence tumor development by providing novel markers for breast cancer prognosis and by improving our understanding of disease onset and progression [118,119,120]. However, most of the current evidence has been derived from 2D culture systems. With the advent of 3D cultures, differential patterns of DNA methylation, histone modifications, chromatin remodeling, and non-coding transcriptomes have been identified, largely mediated by mechanotransduction. In this context, an intricate interplay between DNA methylation, histone modifications, and non-coding RNAs collectively contributes to gene expression dysregulation and the promotion of oncogenic phenotypes [48].

### 7.1. Changes in DNA Methylation in 3D Culture

DNA methylation occurs at CpG dinucleotides within promoters and is associated with gene expression suppression. It plays a critical role in cancer development and progression [119,121]. Specifically, DNA hypermethylation occurs at the carbon-5 position of cytosine residues in CpG islands or at the borders of tumor suppressor gene promoters, leading to decreased gene expression. Conversely, hypomethylation occurs at oncogene promoters, resulting in increased expression [5,121].

Few studies have directly compared DNA methylation changes between 3D and 2D breast cancer cultures. For instance, an increase in DNA (cytosine-5)-methyltransferase 3B (DNMT3B) activity has been reported in 3D Matrigel^®^ cultures of MCF-7 and MDA-MB-231 cells, leading to hypermethylation of the *miR-29c* promoter, reduced *miR-29c* expression, and subsequent upregulation of STAT1, which promotes the formation and growth of cell aggregates [119].

Similarly, Li et al. (2016) [122] observed promoter hypomethylation of several Homeobox (*HOX*) genes (*HOXA9*, *HOXB7*, *HOXB13*, *HOXD10*) in MDA-MB-231 and HS-578T cells cultured in Matrigel^®^ compared with monolayers, resulting in increased protein, though not mRNA expression levels. In particular, HOXA9 exhibited a 32% reduction in promoter methylation relative to monolayer cultures, which was associated with increased expression of bromodomain-containing protein 4 (BRD4). This allowed BRD4 to bind to the HOXA9 promoter exclusively under 3D conditions, likely due to reduced tensile stress. Conversely, hypermethylation of the Retinoblastoma-like protein 1 (RBL1) promoter has been observed in 3D Matrigel^®^ cultures of MCF-7 cells; however, following radiotherapy, promoter hypomethylation occurred, conferring radioresistance only in the 3D-cultured cells compared to those in 2D [123,124].

In contrast, another study reported no significant differences in DNA methylation patterns between 3D and 2D cultures of MCF-7 on alginate scaffold, although transcriptional profiling revealed differential expression of several signal transduction genes, including those from the Mitogen-Activated Protein Kinase (MAPK) and Neurogenic locus notch homolog (NOTCH) pathways [121]. Similarly, in spheroids derived from primary breast tumor tissues, no significant methylation differences were observed for SBDS ribosome maturation factor (SBDS) and transmembrane serine protease 2 (TMPRSS2) genes between 3D and 2D cultures, while the paired box 5 (PAX5) gene showed differential methylation without an assessed biological effect [5].

The available data suggest that 3D culture conditions can induce distinct alterations in DNA methylation patterns compared to conventional 2D cultures. However, the limited amount of evidence currently available prevents the identification of conserved methylation signatures across different 3D culture systems. Therefore, future epigenetic research should integrate comprehensive methylome profiling with detailed biomechanical characterization of 3D matrices to enhance our understanding of the complex regulatory networks underlying breast cancer biology.

### 7.2. Modification of the Histone Code in 3D Culture

Gene expression patterns are determined by chromatin organization, which affects cell fate and differentiated function [63]. This chromatin organization is regulated by various post-translational modifications at the amino-terminal ends of histones, including acetylation, phosphorylation, methylation, and ribosylation, generating a dynamic chromatin state and leading to differential accessibility of transcription factors [63,124]. In a study using HMT-3522-S1 and T4-2 cells cultured in Matrigel^®^ and polyHEMA, both cell lines exhibited global histone deacetylation and increased chromatin condensation compared to 2D culture, leading to aggregate formation independently of the 3D culture methodology. These changes were associated with actin microfilament polymerization, modifying cellular morphology [8,63].

On the other hand, in spheroids derived from the MCF-10A cell progression model and from TNBC breast cancer cell lines (BT-20, CAL-51, HCC70, HCC1937, HCC1806, HS-578T, and MDA-MB-157), a functional genomics approach was applied by silencing the 200 most frequently mutated genes in breast cancer. In this study, the inactivation of the histone acetyl transferase CREB-binding protein (CREBBP) was associated with hypoxia and nutrient depletion compared to observations in 2D cultures. This led to an increase in genomic alterations of the catalytic subunit of phosphatidylinositol 3-kinase (PI3K) and Phosphatase and tensin homolog (PTEN), as well as a 68% reduction in the expression of histone deacetylases (HDAC) and histone acetyltransferases (HAT) [60]. In MCF-7 and T47D spheroids, a higher number of tumor-associated domains (TADs) was observed in 3D cultures compared to monolayer cultures, although the overall number of chromatin domains did not change significantly [46,125]. Interestingly, novel genes capable of forming chromatin loops specifically in 3D cultures were identified within the Hippo signaling pathway, contributing to endocrine therapy resistance [125].

Furthermore, in MDA-MB-231 cells embedded in Matrigel^®^, increased sumoylation of the SnoN protein was observed, enhancing its interaction with HDAC1 and histone acetylase p300. This promoted histone deacetylation compared to 2D cultures, in addition to chromatin remodeling driven by metabolic heterogeneity, which was associated with EMT [126,127]. While in the MCF-10A^HER2^ tumor progression model, which exhibits Doxorubicin-induced HER-2 overexpression and is cultured in Matrigel^®^, malignant transformation was shown to trigger the activation of tyrosine kinases that subsequently activate FAK. This process was accompanied by the enrichment of histone activation marks (H3K4me3, H3K27ac, and H3K9ac) and the reduction of repressive marks (H3K27me3 and H3K9me3) at promoters bound to DExD-box helicase 21 (DDX21), leading to changes in the accessibility of genes associated with cell adhesion, proliferation, and morphogenesis, effects observed only in 3D cultures [124]. Also, it has been reported that in T47D cells embedded in Matrigel^®^, there was a decrease in H3K27ac and H3K18ac marks, along with a 20% increase in super-enhancer presence. This effect was associated with laminin in the scaffold, which activated LATS1 kinase, which could not be observed in the 2D culture. Additionally, regions with the greatest accessibility in 3D cultures were enriched with CCCTC-binding factor (CTCF), Transcriptional Enhanced Associate Domain (TEAD), fork head box A1 (FOXA1), and PR motifs, leading to an increase in the number of hormone-regulated genes [9].

Using HDAC inhibitors such as Panobinostat and CUDC-907, 2D cultures of breast cancer cell lines showed no significant effects after exposure to the inhibitors, unlike what was observed in TNBC-PDTO organoids embedded in Matrigel^®^, where growth was suppressed. These findings indicate that histone deacetylation plays a crucial role in the formation of 3D structures and produces selective effects on tumor cells compared to the non-tumorigenic MCF-10A cell line [48].

On the other hand, in MDA-MB-231 cells embedded in PEG-fibrinogen and PEG-silk fibroin hydrogels with varying matrix stiffness, stiffness was assessed through shear storage modulus measurements and was shown to exert an inverse relationship with the acetylation of histones H3 and H4 in both scaffold types compared to 2D cultures, promoting cyclin D expression and increasing proliferation [92]. Similarly, in DU4475 cells cultured in 3D within Matrigel^®^ and fibrin gels, a decrease in the catalytic subunit of Enhancer of Zeste Homolog 2 (EZH2) of the Polycomb 2 complex was observed, leading to reduced levels of the H3K27me3 mark on chromatin relative to their monolayer counterparts [75]. Another example is the non-histone chromatin structural protein High Mobility Group Box 1 (HMGB1), which is involved in genome repair, transcription, and stability. Table 5 summarizes the chromatin changes associated with 3D culture.

Taken together, these findings suggest that histone modifications, particularly deacetylation, exhibit a conserved pattern across different 3D culture models of breast cancer, seemingly supporting the maintenance of the structural and functional integrity of architecture. Consequently, HDAC inhibitors appear to be promising therapeutic agents capable of altering this organization and selectively targeting tumor cells. However, further experimental and clinical studies are needed to validate these observations and fully elucidate the role of histone deacetylation in the pathophysiology of breast cancer.

## 8. Modification in the Expression of Non-Coding RNAs in 3D Cultures

In addition to modifications at the DNA and chromatin levels, epigenetic reprogramming also involves changes in the expression of non-coding RNAs. These RNA molecules are not translated into proteins but play a crucial role in regulating gene expression [128]. Non-coding RNAs can be grouped into short transcripts (20–30 nucleotides) such as miRNAs and piRNAs; intermediate-sized transcripts (30–200 nucleotides) such as pnRNAs and pnoRNAs; and long transcripts (>200 nucleotides) such as lncRNAs, among others [129].

### 8.1. Alterations in circRNAs Programs

The circRNAs are a class of endogenous RNAs generated through RNA splicing and ligation, forming continuous, covalently closed loops without a 5′ cap or 3′ polyadenylated tail. These loops act as “sponges” for miRNAs and RNA-binding proteins (RBPs), inhibiting their function [130].

Few studies have analyzed circRNAs expression in breast cancer using 3D cultures. Studies from Talhouk’s laboratory compared 3D cultures in Matrigel^®^ and polyHEMA using the HMT-3522 S1 cell tumor transformation model, revealing that reduced expression of Connexin 43 (Cx43) induced delocalization of β-catenin, resulting in the loss of apical polarity exclusively in polyHEMA spheroids compared with Matrigel^®^ cultures [66,131]. Subsequently, to further explore epigenetic reprogramming during malignant transformation, they analyzed circRNA expression in HMT-3522 S1 cells with Cx43 silencing cultured in Matrigel^®^, identifying the overexpression of *hsa_circ_0001568*, *hsa_circ_405443*, and *hsa_circ_0039238*, along with the underexpression of *hsa_circ_0001072* and *hsa_circ_0084765*. They also reported that the differential expression of these circRNAs was associated with the upregulation of *miR-99a-3p*, *miR-8072*, *miR-203a-3p*, *miR-520g-5p*, *miR-182-5p*, *miR-511-5p*, *miR-653-5p*, and *miR-600*, and the downregulation of *miR-520g-3p*, *miR-520h*, and *miR-3960* [66,130,131,132]. These findings are promising for the establishment of a ceRNA network associated with tumor transformation; however, this remains one of the few studies that have investigated circRNA expression under 3D culture conditions.

### 8.2. Modification in microRNA Expression Profiles

The miRNAs are small non-coding RNA molecules that bind to target mRNAs through sequence complementarity, reducing translation or inducing degradation, thereby regulating gene expression [2,10]. Among non-coding RNAs, miRNAs are the most studied group in 3D cultures, and comparisons are frequently made with their expression in 2D systems (Table 6).

In MCF-7 cell spheroids, several miRNAs have been reported to display differential expression patterns that impact key oncogenic processes. Vesuna et al. (2021) [133] demonstrated that *miR-22* targets Forkhead box protein O1 (FOXO1) and PTEN, promoting proliferation, EMT, and resistance to apoptosis, but only in 3D cultures. Other miRNAs whose expression is decreased in MCF-7 cells cultured in 3D vs. 2D were *miR-31-5p*, *miR-221-3p*, *miR-19b-3p*, *miR-130a-3p*, *miR-297-5p*, *miR-181a-2-3p*, *miR-18a-5p*, *miR-18a-3p*, *miR-1306-3p* and *miR-1244*, which are associated with the regulation of tumor protein p73 (TP73), tumor protein p63 (TP63), tumor protein p53 (TP53), Argonaute RISC catalytic component 2 (AGO2), B-Raf proto-oncogene, serine/threonine kinase (BRAF) and hypoxia-inducible factor 1-alpha (HIF1A), influencing development, signaling, morphology and cell maintenance [59]. Similarly, the overexpression of *miR-323a*, *miR-369*, *miR-30e*, *miR-483*, *miR-6501-5p* and *miR-627-5p*, together with the downregulation of *miR-4787*, *miR-1468*, *miR-130a*, *miR-4446*, *miR-1250*, *miR-127*, *miR-301a*, *miR-301a-3p*, *miR-483-3p*, *miR-1468-5p* and *miR-4787-3p* has been linked to the modulation of MYC proto-oncogene (MYC), E2F transcription factor (E2F), forkhead box O 3 (FOXO3), blood vessel epicardial substance (BVES) and Rho GTPase activating protein 17 (ARHGAP17), contributing to proliferation and oxidative phosphorylation (OXPHOS), specifically in spheroids [64].

While Boo et al. (2016) [134] described a broader set of modulated miRNAs in MCF-7 cell spheroids compared to monolayer cultures, including the overexpression of: *miR-4492*, *miR-410*, *miR-4532*, *miR-381*, *miR-127*, *miR-411*, *miR-1246*, *miR-409*, *miR-493*, *miR-4508*, *miR-143*, *miR-126*, *miR-1291*, *miR-370*, *miR-136*, *miR-145*, *miR-211*, *miR-378h*, *miR-369*, *miR-99a*, *miR-4485*, *miR-654*, *miR-499a*, *miR-7641-1*, *miR-7641-2* and *miR-153-2* and, conversely, the underexpression of: *miR-4448*, *miR-221*, *miR-27b*, *miR-125b-1*, *miR-760*, *miR-1296*, *miR-301a*, *miR-365a*, *miR-30c-1*, *miR-let-7f-1*, *miR-4286*, *miR-671*, *miR-26b*, *miR-181a-2*, *miR-615*, *miR-4454*, *miR-130b*, *miR-193b*, *miR-let-7b*, *miR-26a-2*, *miR-155*, *miR-454*, *miR-423*, *miR-16-2*, *miR-191*, *miR-92b*, *miR-628*, *miR-424*, *miR-3074*, *miR-30b*, *miR-744*, *miR-100*, *miR-92b*, *miR-99b*, and *miR-7977*. These alterations converge on the regulation of WNT and MAPK pathways, mediating drug resistance, actin cytoskeleton organization, and metabolic processes [134]. Finally, Azzarito et al. (2022) [135] reported the downregulation of *miR-193a-3p* in MCF-7 spheroids, associated with the regulation of endothelial cell proliferation in response to conditioned media.

Similarly, overexpression *miR-323a*, *miR-369*, *miR-30e*, *miR-483* and *miR-4446-3p* has been reported in HCC1395 cell spheroids, while *miR-301a-3p*, *miR-483-3p*, *miR-1468-5p*, and *miR-4787-3p* were found to be downregulated, which is associated with the regulation of proliferation and OXPHOS, was observed only in 3D-cultured cells [64]. Furthermore, overexpression of *miR-22* in MDA-MB-231 cell spheroids has been shown to induce proliferation and resistance to apoptosis, but not in monolayer cultured cells [133].

In MCF-7 cell mammospheres, Naso et al. (2024) [136] reported that the decrease in *miR-218-5p* expression is more pronounced compared to 2D cultures, leading to increased Parkin protein expression and the induction of mitophagy in response to doxorubicin treatment. In addition, analysis of the reported data revealed the overexpression of *miR-940*, *miR-612*, *miR-22* and *miR-124-2*, in contrast to the suppression of *miR-503* and *miR-99a-let-7c*. Furthermore, mammosphere formation by MCF-7 and MDA-MB-436 cells cultured on Poly-HEMA-coated plates was reduced after exposure to compound 11PS04, which inhibits *miR-21* and upregulates *miR-205*, thereby promoting programmed cell death protein 4 (PDCD4) overexpression and Zinc Finger E-Box Binding Homeobox 1 (ZEB1) downregulation, an effect that was not observed in 2D cultured cells [137].

Furthermore, in mammospheres derived from breast cancer patients of the luminal A and B subtypes, downregulation of *miR-335*, *miR-143*, *miR-140-5p*, *miR-140-3p*, *miR-145*, *miR-181a-2*, *miR-1*, *miR-543*, and *miR-24-1*. These alterations were associated with the modulation of targets such as BCL2 apoptosis regulator (BCL2), GLI family zinc finger 3 (GLI3), Kruppel-like factor 4 (KLF4), NOTCH, Mothers against decapentaplegic homolog 2 (SMAD2), SRY-box transcription factor 4 (SOX4), Sp1 transcription factor or Specificity Protein 1 (SP1), Bone Morphogenetic Protein 2 (BMP2), transforming growth factor beta receptor 1 (TGFBR1), Kirsten rat sarcoma viral oncogene homolog (KRAS), Receptor tyrosine-protein kinase erbB-4 (ERBB4), Vascular Endothelial Growth Factor A (VEGFA), Kruppel-like factor 5 (KLF5), RAS p21 protein activator 1 (RASA1), Cadherin-2 (CDH2), fibroblast growth factor 1 (FGF1), mitogen-activated protein kinase kinase kinase 7 (MAP3K7), Vascular endothelial zinc finger 1 (VEZF1) and ZFP36 ring finger protein like 1 (ZFP36L1), when compared with differentiated adherent cells [67]. From these data, *miR-145* and *miR-543* were identified as potential master regulators, since they control the expression of nearly half of the identified targets.

On the other hand, in two tumor progression models using the MCF-10A.B2 cell line, which overexpresses ERBB2, and the MCF-10A.B2Cas cell line, which overexpresses the p130Cas protein, differential miRNA expression was observed in 3D cultures embedded in Matrigel^®^ compared to 2D cultures. Specifically, 40 invasion-associated miRNAs were identified, including the downregulation of *miR-221*, *miR-27a*, *miR-34b*, *miR-33b*, *miR-16*, *miR-15a*, *miR-1308*, *miR-224*, *miR-181a*, *miR-30e*, *miR-509-3p*, *miR-1295*, *miR-27b*, *miR-23b*, *miR-513b*, *miR-1290*, *miR-149*, *miR-378*, *miR-34c-5p*, *and miR-455-3p*, and the upregulation of *miR-200b*, *miR-222*, *miR-424*, *miR-193a-3p*, *miR-361-3p*, *miR-193a-5p*, *miR-125b*, *miR-221*, *miR-210*, *miR-100*, *miR-99a*, *miR-29b*, *miR-132*, *miR-31*, *miR-let-7i*, *miR-200c*, *miR-365*, *miR-425*, *miR-let-7b*, and *miR-17* [72]. The regulation of these miRNAs affects amino acid synthesis, cell motility, migration, and angiogenesis.

In another study using the HMT-3522 tumor progression cell system, Matrigel^®^ culture was shown to inhibit integrin β-1 and alter the miRNA expression profile compared to 2D culture [130,132,141]. Specifically, the upregulation of circRNAs was associated with a reduction in *miR-362-5p*, *miR-22-3p*, *miR-532-3p*, *miR-520g-3p*, *miR-520h*, *miR-3960*, *miR-874-3p*, *miR-139-5p*, *miR-145-5p*, *miR-100-5p*, *miR-125b-5p*, *miR-200c-3p*, and *miR-3960*. This regulation inhibited the expression of PDCD4, FOXO1, the ubiquitin protein ligase E3 containing beta-transducin repeats (BTRC), and transforming growth factor beta 2 (TGF-β2) [130,132,141]. Collectively, the differential regulation of these miRNAs was associated with the modulation of cell proliferation, migration, viability, and polarity [130,132,141].

In organotypic cultures of MCF-7 cells embedded in Matrigel^®^, a decrease in the expression of *miR-29c* and *miR-29b-3p* was observed, which enabled the formation of organotypic structures and promoted chemoresistance through MYC overactivation, positively regulating BCL-2, PI3K, and AKT serine/threonine kinase 2 (AKT2) [119,123]. These alterations contributed to enhanced radioresistance, angiogenesis, proliferation, invasion, and migration in these 3D cultures.

In MDA-MB-231 cells embedded in Matrigel^®^, a decrease in the expression of *miR-1308*, *miR-301a*, *miR-381*, *miR-140-3p*, *miR-1280*, *miR-18a*, *miR-29c* and *miR-5683* was observed, whereas an increase in *miR-1246*, *miR-654-5p*, *miR-663*, *miR-1469*, *miR-1915*, *miR-762*, *miR-149*, *miR-1826*, *miR-1908*, *miR-575*, *miR-1231*, *miR-1975*, *miR-1977*, *miR-1978*, *miR-638*, *miR-1275*, *miR-150*, *miR-1974*, *miR-128-1*, *miR-193a-5p*, *miR-let-7g* and *miR-27b* [119,138,139]. This modulation of miRNA expression was associated with the regulation of DNMT3B, FOXO1, tissue inhibitor of metalloproteinase 3 (TIMP3), STAT1, ATP citrate lyase (ACLY), Rac GTPase-activating protein 1 (RACGAP1), adenylate kinase 4 (AK4), mitochondrial ribosomal protein L51 (MRPL51), Cytochrome b5 type B (CYB5B), makorin ring finger protein 1 (MKRN1), transmembrane protein 230 (TMEM230), nucleoporin 54 (NUP54), anaphase-promoting complex subunit 13 (ANAPC13), Phosphoglycerate Mutase 1 (PGAM1), and superoxide dismutase 1 (SOD1), thereby impacting aggregate formation, the characteristic stellate morphology of this cell line in 3D culture, as well as its metabolism and invasive capacity.

In other TNBC breast cancer models, such as BT-549 and MDA-MB-436, compared to 2D culture, Matrigel^®^ culture induced a reduction in *miR-29c* and *miR-5683*, which was partially linked to DNMT3B-mediated promoter methylation and the regulation of metabolism through the modulation of ACLY, RACGAP1, AK4, MRPL51, CYB5B, MKRN1, TMEM230, NUP54, ANAPC13, PGAM1, and SOD1 [119,138].

In a different approach, using Geltrex^®^ as a scaffold in SK-BR-3 cells, 39 differentially expressed miRNAs were identified compared to 2D cultures. Among these, *miR-6529-5p*, *miR-122-5p*, *miR-410-3p*, *miR-409-3p* and *miR-369-3p* were upregulated, whereas *miR-449c-3p*, *miR-449b-3p*, *miR-3689a-3p*, *miR-449a* and *miR-1247-5p* were downregulated. These miRNAs target 70 mRNAs that were downregulated and are associated with the regulation of morphogenesis, WNT signaling, MAPK signaling, and Advanced glycation end-product-Receptor for advanced glycation end-product (AGE-RAGE) pathways [2]. Meanwhile, on 3D cultures of BT-474 cells, increased expression of *PIP4K2B*, *FAM3B*, *TMSB4XP1*, *CST1*, *TMSB4X*, *RAD51C*, *AFF3*, *UPK1A*, *ACSF2*, *and TMEM150C* was reported, whereas *TMSB4XP6*, *PCGF2*, *ZNF254*, *MDK*, *PRPS2*, *CST4*, *LRRN1*, *TFF3*, *VN1R53P*, and *NPY1R* were downregulated, promoting glycolysis and OXPHOS in organotypic cultures [23].

In the case of Cultrex^®^, Taylor et al. (2013) [82] reported in the 4T1 murine progression system and MDA-MB-231 cells that the stiffness of the ECM alters miRNA expression partially by regulating the TGF-β response. Specifically, *miR-181a* was necessary, but not sufficient, to promote tumor invasion in response to TGF-β by enhancing phosphorylation of SRC, AKT, and ERK 1/2, without affecting proliferation [10]. Furthermore, using PCL as a scaffold, MDA-MB-231 cells exhibited down-expression of *miR-1908*, *miR-7974*, *miR-671-5p*, *miR-769-3p*, *miR-4750-5p*, *miR-5587-3p*, *miR-0386-3p*, *miR-0415-3p*, *miR-1268b*, *miR-877-3p*, *miR-139-3p*, *miR-4758-3p*, *miR-1915-5p* and *miR-3940-5p*, whereas *miR-210*, *miR-27a*, *miR-146a*, *miR-619-5p*, *miR-1260*, *miR-1237f* and *miR-1538* were upregulated, compared to monolayer cultures. These miRNAs are involved in pluripotency and metastasis [10].

Based on the available data, specific miRNA expression profiles can be associated with distinct molecular subtypes of breast cancer. For instance, the luminal A subtype typically overexpresses miRNAs involved in proliferation, survival, and chemoresistance, whereas tumor-suppressor miRNAs are downregulated, leading to activation of the PI3K/AKT signaling pathway. In the HER-2+ subtype, miRNAs regulating proliferation and ECM remodeling are upregulated, thereby promoting activation of the MAPK and WNT signaling pathways. Conversely, the TNBC subtype exhibits overexpression of miRNAs associated with EMT, invasion, pluripotency, and oxidative metabolism, along with the downregulation of miRNAs related to cell adhesion. This expression pattern contributes to the activation of signaling cascades such as PI3K/AKT, FOXO1, Nuclear Factor kappa-light-chain-enhancer of activated B cells (NF-κB), and TGF-β. However, it is important to note that both the scaffold composition and the analytical platform significantly contribute to experimental heterogeneity, underscoring the need for studies that compare identical cell lines across different 3D culture systems using standardized platforms.

Additionally, miRNA expression is influenced by the type of 3D culture. Spheroids predominantly regulate miRNAs involved in chemoresistance, metabolism, and proliferation, whereas cultures in Matrigel^®^ or Geltrex^®^ show enrichment of miRNAs controlling morphogenesis and activation of WNT and MAPK signaling. Together, these findings demonstrate that miRNA expression in breast cancer is shaped not only by molecular subtype but also by the scaffold type used in 3D culture, highlighting the interplay between tumor biology and microenvironmental cues. Despite variations in miRNA expression, it is possible to identify shared regulatory candidates across different scaffold types and cell lines (Table 7). These candidates should be further validated through batch correction and cross-validation across multiple 3D culture methodologies.

### 8.3. Deregulation of LncRNAs Landscapes

Currently, 3D culture systems have been used to screen gene expression patterns [140], allowing the identification of transcriptomic differences between tumor cells cultured in 2D versus 3D systems. One important class of transcripts, lncRNAs, regulates gene expression [17,23]. The lncRNAs are transcripts longer than 200 nucleotides that are not translated into proteins and can act as decoys, guides, scaffolds, and miRNA sponges, thereby regulating apoptosis, cell cycle, and metastasis. However, its role in 3D cultures remains to be fully explored [23,97,142].

In MCF-7 cell spheroids compared with 2D cultures, downregulation of *LINC00052*, *LINC01235*, *PURPL*, *LINC02233*, *LINC00869*, *LINC00623*, *LINC01239*, *LINC00492*, *LINC02067*, *LINC02367*, *FCGR1B-1:1*, *AC006372.1-2:1*, *CAMK1G-1:2*, *TMEM51-AS2*, *RP11-1070N10.3.1*, *PLGLB2-1:7*, *DUOXA1-1:1*, *DBN1-2:4*, *WRNP1-2:16*, and *LMNTD2* has been reported. This suppression correlates with the upregulation of genes involved in cholesterol, stearate, and mevalonate metabolic pathways, such as Transforming Growth Factor Beta 3 (TGF-β3), SRY-Box Transcription Factor 9 (SOX9), Frizzled Class Receptor 4 (FZD4), WNT Family Member 4 (WNT4), SRY-Box Transcription Factor 12 (SOX12), TGF-β2, Protein Phosphatase 2 Regulatory Subunit Bβ (PPP2R2B), Cyclin-D1 Binding Protein 1 (CCNDBP1), TGF-β1, Mevalonate Diphosphate Decarboxylase (MVD), 3-Hydroxy-3-Methylglutaryl-CoA Synthase 2 (HMGCS2), Acetyl-CoA Acetyltransferase (ACAT), TRAF Family Member-Associated NF-κB Activator (TANK), and Poly(ADP-ribose) Polymerase Family Member 9 (PARP9), thereby promoting proliferative programs [17,143]. Conversely, overexpression was observed for *LINC01790*, *LINC00362*, *LINC01213*, *LINC00996*, *LINC02380*, *LINC01983*, *LINC01310*, *LINC00299*, *LINC01794*, *LINC01258*, *LINC00467*, *MCM3AP-AS1*, *PVT1*, *H19*, *MALAT1*, *GAS5-AS1*, *TP53TG1*, *MEG3*, *TUG1*, *ZFAS1*, *CAPN7-1:2*, *CPEB4-6:1*, *OSBPL6-2:6*, *IGSF5-1:1*, *MLN-1:2*, *RIM-KLB-1:1*, *LGALS14-1:2*, *TRIM43-1:1*, *SCYL1-1:4*, and *TCL1B-1:1*, converging on MYC, P53, mTOR, and HIF1A signaling [17,143]. Together, these lncRNA expression changes contribute to alterations in cell-cycle progression and OXPHOS.

On the other hand, using a tumor progression model with MCF-10A cells carrying activated T24-HRAS and xenograft selection, subclones representing various stages of mammary tumorigenesis were cultured in Matrigel^®^. This allowed the comparison of lncRNA expression between metastatic-stage cells and premalignant lesions, revealing down-expression of *AC009299.3*, *RP11-463O9.5*, *RP3-460G2.2*, *RP1-124C6.1*, *RP11-74E22.3*, *LINC00640*, *RP11-326C3.7*, *RP11-1000B6.5*, *AC098617.1* and *RP4-737E23.2*, while *LINC00973*, *AC019172.2*, *LINC02154*, *LINC00431*, *RP11-24N18.1*, *RP11-217E22.5*, *RP11-879F14.2*, *RP11-3L8.3*, *RP11-626H12.2*, *LINC01705* were overexpressed [129]. This caused a decrease in the expression of PDCD4, Forkhead Box C2 (FOXC2), Solute Carrier Family 2 Member 9 (SLC2A9), Cyclin D1 Binding Protein 1 (CCNDBP1) and Intercellular Adhesion Molecule (ICAM), modifying the cell cycle and EMT during malignant transformation.

Furthermore, in organotypic cultures on Matrigel^®^ using MCF-7 cells resistant to tamoxifen (LCC2) and to fulvestrant and tamoxifen (LCC9), one of the resistance mechanisms was reported to be mediated by the overexpression of the lncRNA *H19*, through the activation of the NOTCH and MET Proto-Oncogene, Receptor Tyrosine Kinase (C-MET) signaling pathways, a specific resistance mechanism observed only in 3D aggregates [96]. In MDA-MB-231 and HS-578T cells cultured on Matrigel^®^, the *HOTAIR-N* variant was overexpressed compared to the 2D culture, an effect mediated by the activation of integrin α2 and SRC, which promotes invasive growth in organotypic cultures [144]. Similarly, García-Hernández et al. (2024) [97], it was observed that *AFAP1-AS1* overexpression is necessary for the formation of 3D cultures of HS-578T Matrigel^®^ cells under hypoxic conditions.

While in Matrigel^®^ cultures of murine tumors derived from the MMTV-PyMT line (luminal B subtype) and the MMTV-Neu-NDL line (HER-2+ subtype), overexpression of *Rik-203*, *Gm16025*, *A330074K22Rik*, *Gm13387*, *RP23-437C24.2*, and *RP23-327I19.1* was reported, underscoring the regulatory role of the androgen receptor and P63 during malignant transformation [77]. In an “on-top” BT-474 culture model embedded in Geltrex^®^, differential expression of 473 lncRNAs was observed in 3D versus 2D cultures, with 290 overexpressed and 183 suppressed [23]. Among these, the most overexpressed lncRNAs were *RP11-20F24.2*, *UCA1*, *LASP1NB*, *CTD-2566J3.1*, and *LINC00847*, while the most repressed were *XIST*, *CYTOR*, *LINC00857*, *MIR4435-2HG*, which were associated with proliferation, migration, invasion, and metastasis. These lncRNAs were linked to Epidermal Growth Factor Receptor (EGFR) and TGF-β signaling pathways, influencing glycolysis and mitochondrial respiration. *CTD-2566J3.1* and *LINC00847* acted as master regulators of processes including estrogen response, apical junctions, and drug resistance, through the *CTD-2566J3.1*/GINS2 and *LINC008477*/FOXA1 axes [23].

Since few studies and breast cancer models have compared lncRNA expression patterns, it is not yet possible to identify a signature associated either with a specific 3D culture system or with the molecular subtype of the disease. Table 8 summarizes the differentially expressed lncRNAs in breast cancer cells in relation to the type of 3D culture compared to two-dimensional culture.

## 9. Limitations and Perspectives

In general, the data described in this review demonstrate that different 3D culture methods induce distinct variations in tumor cell behavior, largely determined by the presence or absence of ECM-mimicking components. As detailed throughout the review, these differences at the protein, transcriptomic, and epigenetic levels arise from the activation of signaling pathways regulated by mechanotransduction. This highlights that scaffold-based systems represent an evolutionary refinement of 3D culture models, aiming to achieve a higher degree of similarity to patient tumors. However, this goal has not yet been fully realized, as several limitations continue to restrict their widespread use and standardization.

The first limitation is associated with the nature and origin of the biomaterial. Protein composition varies substantially between matrices and even between batches of the same commercial product. Collagen and laminin are critical for modulating pathways such as WNT, MAPK, and PI3K/AKT, which subsequently influence the activity of epigenetic enzymes like DNMT3B. Thus, reporting detailed information, including manufacturer and batch number, could improve the reproducibility of studies and allow comparisons of matrix composition across investigations.

A second challenge concerns scaffold stiffness and pore size. As demonstrated in collagen and GelMA-based models [103,112], increases in elastic modulus correlate with reduced histone acetylation through ILK/FAK/RhoA signaling. Simultaneously, matrix porosity shapes the spatial distribution of cells, nutrients, and oxygen, providing an alternative regulatory mechanism through altered glycolytic metabolism and HDAC inhibition [145]. These biomechanical properties, therefore, influence cellular plasticity and therapeutic responses. Consequently, reporting physicochemical fabrication parameters is essential to prevent epigenetic variability between experiments that are expected to be equivalent.

Furthermore, another current limitation for identifying conserved epigenetic signatures in 3D systems lies in the scarcity of epigenomic data, as most studies focus on transcriptome comparisons between 3D cultures and tumor tissues. Therefore, emerging technologies offer the opportunity to generate complete profiles of the non-coding transcriptome of tumor cells. In addition, the findings described in this review suggest further investigation into the identification of chromatin patterns as a potential treatment biomarker.

Additionally, it is important to recognize that many of the cell lines commonly used in these studies display limited similarity to human tumors, largely due to mutation accumulation and copy-number alterations acquired during long-term passaging [35,146]. Moreover, the predominant use of monocultures fails to recapitulate the complexity of the tumor microenvironment, where intricate interactions occur among tumor cells, fibroblasts, adipocytes, endothelial cells, and macrophages. From this perspective, tumors PDTOs represent an ideal model for defining transcriptomic and epigenetic landscapes. Their ability to preserve both the molecular and structural heterogeneity of tumors makes them a promising platform for therapeutic prediction and for identifying clinically applicable biomarkers.

On the other hand, to strengthen the reproducibility and precision of three-dimensional culture studies, we propose implementing a standardized experimental framework that includes detailed reporting of scaffold origin, batch number, biochemical composition, and polymerization conditions, together with the systematic measurement of key biophysical parameters—such as stiffness, elastic modulus, and pore size—both before and after culture. We further recommend conducting parallel comparisons between 2D systems and multiple 3D platforms while controlling variables such as cell density, aggregate size, and microenvironmental conditions related to oxygen and pH gradients. Integrating multi-omics analysis including DNA methylation profiling, histone modification mapping, and the expression of miRNAs, lncRNAs, and circRNAs—will better capture the combined influence of mechanotransduction and scaffold architecture on cellular regulation. Additionally, incorporating functional controls, such as epigenetic inhibitors or stiffness modulators, along with bioinformatic pipelines that account for batch effects and enable the integration of transcriptomic and epigenomic data, will facilitate the causal linking of the physicochemical properties of 3D microenvironments with epigenetic variation. Altogether, these strategies will enhance comparability across studies and support the identification of robust biomarkers associated with tumor behavior in three-dimensional systems.

## 10. Conclusions

The 3D breast cancer cultures represent superior models to traditional 2D systems for studying tumor heterogeneity, as they more faithfully reproduce tissue architecture. These models allow for detailed investigation of cancer biology, including the interaction between tumor cells and their microenvironment through key signaling pathways such as PI3K/Akt, MAPK, and TGF-β, which exhibit bidirectional feedback with specific epigenetic modifiers, collectively regulating phenotype, proliferation, pluripotency, and chemoresistance. Moreover, the composition and stiffness of scaffolds directly influence intracellular signaling and epigenetic reprogramming through mechanotransduction, emphasizing the role of the physical microenvironment in cellular plasticity. Despite these advantages, variability between culture systems and matrix types limits reproducibility and complicates the identification of universal therapeutic targets, underscoring the need for standardized methodologies to facilitate translational applications across different breast cancer subtypes. Nevertheless, current evidence suggests that histone modification appears to be a conserved biomarker across different 3D culture systems and molecular subtypes of breast cancer, primarily through histone deacetylation. Therefore, HDAC inhibitors could emerge as a potential therapeutic target. In contrast, the epigenetic profiles of non-coding RNAs appear to depend on the specific 3D culture method used. Specifically, *miRNA-22*, *miR-301a*, and *miR-181a* exhibit the same type of regulation across different 3D culture systems, emerging as potential treatment targets.

## Figures and Tables

**Table 1:** Three-Dimensional culture morphology of breast cancer cells.

Type of Morphology	Molecular Subtype	Cell Line	Gene Signature	Reference
**Mass** 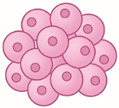	Pre-invasive	T4-2	↑ ERBB2	[29]
	Luminal A	MCF-7		[31]
		T47D		
	Luminal B	BT-483		[29]
	Her-2+	BT-474		
	TNBC	BT-20		[12]
		HCC70		[29]
**Round** 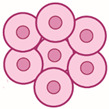	Non-tumorigenic	MCF-12A	—	[29]
	Pre-invasive	S1		
	Luminal A	MDA-MB-415		
		MPE-600		
	TNBC	HCC1500		
**Grape-like** 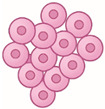	Luminal A	ZR-75-B	↑ ERBB2	[29]
	Luminal B	CAMA-1		
		MDA-MB-361		
		ZR-75-1		
	Her-2+	AU565		
		SK-BR-3		
		UACC-812		
		SK-BR-3		
	TNBC	MDA-MB-453		
		MDA-MB-468		
**Star-like** 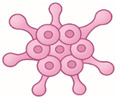	TNBC	BT-549	↑ PPARG ↑ FADS1 ↑ PTGER4 ↑ PARG/↑ PALM2	[29]
		HS-578T		
		MDA-MB-231		
		MDA-MB-436		

Erythroblastic Oncogene B2 (ERBB2); Peroxisome Proliferator-activated Receptor Gamma (PPARG); Fatty Acid. Desaturase 1 (FADS1); Prostaglandin E Receptor 4 (PTGER4); Poly(ADP-ribose) Glycohydrolase (PARG); Paralemmin (PALM2). ↑ = increase in the expression of the molecule.

**Table 2:** Breast cancer cell lines used in different 3D culture methods.

Methodology	3D Culture Type	Support	Molecular Subtype	Cell Line	Reference
**Scaffold-free**	Spheroids	ULA plates	Non-tumorigenic	MCF-12A	[52]
			Luminal A	MCF-7, T47D, MDA-MB-134-VI, MDA-MB-330, Sum44PE	[33,34,53,54,55]
			Luminal B	ZR-75-1, KAIMRC1	[9,29,56]
			Her-2+	SK-BR-3, BT-474, JIMT1	[29,53,57,58]
			TNBC	MDA-MB-231, MDA-MB-468, BT-20, BT-549, HS-578T, CAL-51, HCC70, HCC1937, HCC1806, MDA-MB-157, SUM1315	[33,34,53,59,60,61]
			PDTO	TU-BcX-2 K1	[62]
		PoliHEMA	Pre-invasive	T4-2	[63]
			Luminal A	MCF-7	[64]
			Luminal B	ZR-75-1	[65]
			Her-2+	BT-474, JIMT1	[58]
			TNBC	HMT-3522 S1, HCC1395, BT-20	[64,65,66]
	Mammospheres	ULA plates	Luminal A	MCF-7, T47D	[33,35,36]
			Luminal B	BT-474, SK-BR-3	[8,67]
			TNBC	HS-578T, SUM149, MDA-MB-468, BT-549, MDA-MB-231	[8,33,35,36,67,68]
	Magnetic levitation	Magnetic nanoparticles	Luminal A	MCF-7	[26,37]
			TNBC	MDA-MB-231, SUM159, HS-578T	[26,37,69]
	Hanging drop	Hanging drop plates	Luminal A	MCF-7, T47D	[26,40,70]
			Her-2+	SK-BR-3, BT-474	[71]
			TNBC	MDA-MB-231, BT-20	[12,70]
**Scaffold-based**	Organotypic cultures	Matrigel^®^/ Collagen	Progression model	MCF-10a.B2, MCF-10A.B2Cas	[72]
			Luminal A	MCF-7, T47D, CAMA-1	[6,7,30,41]
			Luminal B	ZR-75-1	[9]
			Her-2+	SK-BR-3, BT-474, MDA-MB-361, AU565, BT-483, HCC1954	[6,7,9,14,30,36,41]
			TNBC	MDA-MB-231, MDA-MB-436, MDA-MB-453, MDA-MB-468, BT-549, HCC70, HS-578T, HCC1143, SUM149PT, HCC1806, DU4475	[14,30,73,74,75]
	PDTO	Matrigel^®^/ ECM	TNBC	HS-578T, TNBC patient-derived	[31,42,43,76]
		Geltrex™	Luminal A	Luminal patient-derived	[45,76]
		Type I collagen	Her-2+	Her-2+ patient-derived	[76]
	PDX	Matrigel^®^	Luminal B	MMTV-PyMT,	[77]
			Her-2+	BT-474, MMTV-Neu-NDL	[46,47,48,77]
			TNBC	MDA-MB-231	[46,47,48]
	OoC	Microfluidic chip	Luminal A	MCF-7	[47,49,50]
			TNBC	MDA-MB-231	
	3D Bioprinting	Bioink/ Hydrogel	Luminal A	MCF-7, T47D	[6,16,41]
			Luminal B	ZR-75-1	[15]
			TNBC	MDA-MB-231	[6,16,41]

**Table 3:** Breast cancer cells in different types of 3D culture scaffolds.

Scaffold	Molecular Subtype	Cell Line	References
**Matrigel^®^**	Non Tumoral	MCF-10A	[93,94]
	Progression model	MCF-10A-Ras	[95]
	Luminal A	MCF-7, CAMA-1, T47D, MCF7/LCC2, MCF7/LCC9, MDA-MB-134-VI, MDA-MB-330, Sum44PE	[28,30,55,95,96]
	Luminal B	ZR-75-1	[30]
	Her-2+	MDA-MB-361, SK-BR-3, AU565, BT-474, BT-483	
	TNBC	MDA-MB-231, MDA-MB-436, MDA-MB-453, MDA-MB-468, BT-549, HCC70, HS-578T	[6,18,30,96,97]
	PDTO	TU-BcX-2 K1	[62]
**Geltrex^®^**	Non Tumoral	MCF-10A	[18]
	Her-2+	SK-BR-3, BT-474	[23]
	TNBC	HS-578T, SUM1315	[25,82]
**Cultrex^®^**	Luminal A	MCF-7	[18]
	TNBC	MDA-MB-231	[82]
**Type I** **collagen**	Luminal A	MCF-7, MDA-MB-134-VI, MDA-MB-330, Sum44PE	[55,75,98]
	TNBC	MDA-MB-231, DU4475	[98]
**dECM**	Luminal A	MCF-7	[89]
	TNBC	MDA-MB-231, HCC1806, 4T1	[97]
**Freeze-dried** **collagen**	Luminal A	MCF-7	[99]
	TNBC	MDA-MB-231, MDA-MB-468	
**Fibrin Gels**	TNBC	MDA-MB-231, DU4475	[75,100]
**Alginate**	Luminal A	MCF-7	[101]
	TNBC	MDA-MB-231	
**PHB**	TNBC	4T1	[18]
**PCL**	TNBC	MDA-MB-231	[100]
**SCPL**	TNBC	4T1	[18]
**PEG**	Luminal A	PDTO	[102]
	Her-2+	PDTO	
	TNBC	MDA-MB-231, PDTO	[100,102]
**Red FN-silk**	Luminal A	MCF-7	[27,32]
	Her-2+	SK-BR-3	[32]
	TNBC	MDA-MB-231	
**PHA** **polymers**	Luminal A	MCF-7	[18]
	TNBC	MDA-MB-231	
**GelMA**	Luminal A	MCF-7, PDTO	[102,103]
	Her-2+	PDTO	[104]
	TNBC		
**GelAGE**	Luminal A	MCF-7	[103]
**GelSH**	Luminal A	PDTO	[102]
	Her-2+		
	TNBC		
**3D** **bioprinting**	Luminal A	MCF-7	[105]
	TNBC	MDA-MB-231	

**Table 4:** Comparative characteristics of basement membrane extracts used in 3D culture systems.

Characteristic	Matrigel^®^	Geltrex^®^	Cultrex^®^
**Manufacturer**	Corning (formerly BD Biosciences)	Thermo Fisher Scientific (Invitrogen)	R&D Systems/Bio-Techne
**Biological** **origin**	Extract from EHS	Extract from EHS	Extract from EHS
**Main** **composition**	Laminin (~60%), Collagen IV (~30%), Entactin (~8%), Heparan sulfate proteoglycans	Similar composition; more purified and with lower batch variability	Laminin, Collagen IV, Nidogen, Proteoglycans and tumor-free formulations
**Total protein concentration**	8–12 mg/mL (batch dependent)	8–12 mg/mL	3–18 mg/mL, depending on formulation
**Elastic** **Modulus G′**	150–450 Pa (batch and temperature dependent)	Similar (150–400 Pa), better consistency	Controlled: “soft” (~100–200 Pa), “standard” (~300–500 Pa), “stiff” (>1000 Pa)
**Endotoxin** **content**	Variable; typically < 5 EU/mL	Low; <2 EU/mL	Controlled (<1 EU/mL)
**Batch-to-batch reproducibility**	Moderate; batch validation required	Improved reproducibility	High reproducibility (lot-specific certification)
**Limitations**	Batch variability, tumor-derived origin	Lower variability but still animal-derived	Higher cost; less representation in historical literature

**Table 5:** Overview of Histone Modification Dynamics in Breast Cancer 3D Culture Systems.

Culture Method	Molecular Subtype	Cell Line	Platform	No. Cultured Cells	Type Regulation	Target Genes/ Pathways	Functional Effects	Reference
**Spheroids**	Non-tumor	MCF-10A	Mass spectrometry	27,000 cells/mL	Decrease in HDAC and HAT	CREBBP, PI3K and PTEN	Hypoxia	[60]
	Luminal A	MCF-7	Hi-C profiling	25,000 cells/mL	TADs increase	Hippo pathway	Endocrine therapy resistance	[46,125]
		T47D						
	TNBC	BT20, CAL-51, HCC70, HCC1937, HCC1806, Hs578T and MDA-MB-157	Mass spectrometry	27,000 cells/mL	Decrease in HDAC and HAT	CREBBP, PI3K and PTEN	Hypoxia	[60]
		HMT-3522-S1	ChIP assays	4000 cells/cm^2^	Decreased histone deacetylation	--	Actin microfilament polymerization	[8,63]
		T4-2						
**Matrigel^®^**	Non-tumor/ Progression	MCF-10A^HER2^	ATAC-seq	25,000 cells/mL	Increased histone methylation Decreased histone acetylation	FAK and DDX21	Cell adhesion, proliferation, and morphogenesis	[124]
	Luminal A	T47D	ChIP assays	Not specified	Increased H3K27ac and H3K18ac	LATS1 and FOXA1	Hormone Response	[9]
	TNBC	MDA-MB231	Western Blot	6000 cells/mL	Decreased histone deacetylation	SnoN, histone acetylase p300 and HDAC	EMT	[126]
			Smart-seq3D	5000 cells/well	Chromatin remodeling	--	Invasion and metastasis	[127]
		HMT-3522-S1	ChIP assays	4000 cells/cm^2^	Decreased histone deacetylation	--	Actin microfilament polymerization	[8,63]
		T4-2						
		DU4475	Western Blot	200,000 cells/mL	Decreased H3K27me3	EZH2 and HMGB1	DNA repair	[75]
		PDO	Drug Screening	Not specified	Decreased histone deacetylation	HDAC	Antiproliferative	[48]
**PEG-fibrinogen** **PEG-silk**	TNBC	MDA-MB231	Western Blot	65,000 cells/cm^2^	Decreased histone deacetylation	Cyclin D	Proliferation	[92]
**Fibrin Gels**	TNBC	DU4475	Western Blot	200,000 cells/mL	Decreased H3K27me3	EZH2 and HMGB1	Genome repair	[75]

**Table 6:** miRNAs expressed in breast cancer cells in the different 3D culture methods.

Culture Method	Molecular Subtype	Cell Line	Platform	No. Cultured Cells	Increased miRNAs	Decreased miRNAs	Target Genes/ Pathways	Functional Effects	Reference
**Spheroids**	Luminal A	MCF-7	RT-qPCR (*n* = 3)	Not specified	*miR-22*	---	FOXO1 and PTEN	Proliferation, EMT and anti-apoptosis	[133]
			Microarrays (*n* = 3)	160,000 cells/cm^2^	*miR-1290*, *miR-22-5p*, *miR-184*, *miR-487b-3p*, *miR-148a-3p*, *miR-127-3p*, *miR-187-3p*, *miR-379-5p*, *miR-616-3p*, *miR-10a-5p*	*miR-31-5p*, *miR-221-3p*, *miR-19b-3p*, *miR-130a-3p*, *miR-297-5p*, *miR-181a-2-3p*, *miR-18a-5p*, *miR-18a-3p*, *miR-1306-3p*, *miR-1244*	TP73, TP63, TP53, AGO2, BRAF and HIF1A	Cell Development, Proliferation, Cell Signaling, Morphology and Cell Maintenance	[59]
			RNA-seq (*n* = 3)	5000 cells/cm^2^	*miR-323a*, *miR-369*, *miR-30e*, *miR-483*, *miR-6501-5p*, *miR-627-5p*	*miR-4787*, *miR-1468*, *miR-130a*, *miR-4446*, *miR-1250*, *miR-127*, *miR-301a*, *miR-301a-3p*, *miR-483-3p*, *miR-1468-5p*, *miR-4787-3p*	MYC, E2F, FOXO3, BVES and ARHGAP17	Proliferation and OXPHOS	[64]
			RNA-seq (*n* = 3)	150,000 cells/cm^2^	*miR-4492*, *miR-410*, *miR-4532*, *miR-381*, *miR-127*, *miR-411*, *miR-1246*, *miR-409*, *miR-493*, *miR-4508*, *miR-143*, *miR-126*, *miR-1291*, *miR-370*, *miR-136*, *miR-145*, *miR-211*, *miR-378h*, *miR-369*, *miR-99a*, *miR-4485*, *miR-654*, *miR-499a*, *miR-7641-1*, *miR-7641-2*, *miR-153-2*	*miR-4448*, *miR-221*, *miR-27b*, *miR-125b-1*, *miR-760*, *miR-1296*, *miR-301a*, *miR-365a*, *miR-30c-1*, *miR-let-7f-1*, *miR-4286*, *miR-671*, *miR-26b*, *miR-181a-2*, *miR-615*, *miR-4454*, *miR-130b*, *miR-193b*, *miR-let-7b*, *miR-26a-2*, *miR-155*, *miR-454*, *miR-423*, *miR-16-2*, *miR-191*, *miR-92b*, *miR-628*, *miR-424*, *miR-3074*, *miR-30b*, *miR-744*, *miR-100*, *miR-92b*, *miR-99b*, *miR-7977*	WNT and MAPK	Drug resistance, Actin Cytoskeleton and Metabolic process	[134]
			RNA-seq (*n* = 3)	Not specified	---	*miR-193a-3p*	RE	Proliferation	[135]
	TNBC	MDA-MB-231	RT-qPCR (*n* = 3)	Not specified	*miR-22*	---	FOXO1 and PTEN	Proliferation, EMT and apoptosis resistance	[133]
		HCC1395	RNA-seq (*n* = 3)	7500 cells/cm^2^	*miR-323a*, *miR-369*, *miR-30e*, *miR-483*, *miR-4446-3p*	*miR-301a-3p*, *miR-483-3p*, *miR-1468-5p*, *miR-4787-3p*	MYC, E2F, FOXO3, BVES and ARHGAP17	Proliferation and OXPHOS	[64]
**Mammospheres**	Luminal A	MCF-7	Microarrays (*n* = 3)	410,000 cells/cm^2^	*miR-940*, *miR-612*, *miR-22*, *miR-124-2*	*miR-218-5p*, *miR-503*, *miR-99a-let-7c*	PINK1/Parkin	Mitophagy	[136]
			RT-qPCR (*n* = 3)	1000 cells/mL	*miR-21*	*miR-205*	PDCD4 and ZEB1	Apoptosis, EMT, Cell Differentiation and Vascularization	[137]
		PDTO	Microarrays (*n* = 3)	300 cells/cm^2^	*miR-671-5p*, *miR-663*, *miR-146a*, *miR-1224-5p*, *miR-630*	*miR-335*, *miR-143*, *miR-140-5p*, *miR-140-3p*, *miR-145*, *miR-181a-2*, *miR-1*, *miR-543*, *miR-24-1*	BCL2, GLI3, KLF4, NOTCH, SMAD2, SOX4, SP1, BMP2, TGFBR1, KRAS, ERBB4, VEGFA, KLF5, RASA1, CDH2, FGF1, MAP3K7, VEZF1 and ZFP36L1	Apoptosis, EMT, Cell differentiation and Vascularization	[67]
	Luminal B	PDTO	Microarrays (*n* = 3)	300 cells/cm^2^	*miR-671-5p*, *miR-663*, *miR-146a*, *miR-1224-5p*, *miR-630*	*miR-335*, *miR-143*, *miR-140-5p*, *miR-140-3p*, *miR-145*, *miR-181a-2*, *miR-1*, *miR-543*, *miR-24-1*	BCL2, GLI3, KLF4, NOTCH, SMAD2, SOX4, SP1, BMP2, TGFBR1, KRAS, ERBB4, VEGFA, KLF5, RASA1, CDH2, FGF1, MAP3K7, VEZF1 and ZFP36L1	Cell differentiation and Vascularization	[67]
	TNCB	MDA-MB-436	RT-qPCR (*n* = 3)	1000 cells/mL	*miR-21*	*miR-205*	PDCD4 and ZEB1	Apoptosis and EMT	[137]
**Matrigel^®^**	Non- tumor/ Progression	MCF-10A.B2	Microarrays (*n* = 5)	Not specified	*miR-200b*, *miR-222*, *miR-424*, *miR-193a-3p*, *miR-361-3p*, *miR-193a-5p*, *miR-125b*, *miR-210*, *miR-100*, *miR-99a*, *miR-29b*, *miR-132*, *miR-31*, *miR-let-7i*, *miR-200c*, *miR-365*, *miR-425*, *miR-let-7b*, *miR-17*	*miR-221*, *miR-27a*, *miR-34b*, *miR-33b*, *miR-16*, *miR-15a*, *miR-1308*, *miR-224*, *miR-181a*, *miR-30e*, *miR-509-3p*, *miR-1295*, *miR-27b*, *miR-23b*, *miR-513b*, *miR-1290*, *miR-149*, *miR-378*, *miR-34c-5p*, *miR-455-3p*	AKT, ERK 1/2	Invasion	[72]
		HMT-3522 S1	RNA-seq (*n* = 3)	420,000 cells/cm^2^	*miR-99a-3p*, *miR-99a-5p*, *miR-8072*, *miR-203a-3p*, *miR-183-5p*, *miR-520g-5p*, *miR-182-5p*, *miR-511-5p*, *miR-653-5p*, *miR-600*	*miR-362-5p*, *miR-22-3p*, *miR-532-3p*, *miR-520g-3p*, *miR-520h*, *miR-3960*, *miR-874-3p*	PRKCA, GPR50, SNAI2, IGF1R, RAC1, ABL1, VEGFA, SMAD6, PDPK, JAK2 and YAP1	Loss of Cx43, regulation by circRNAs, Migration, Proliferation and FA	[132]
			miRNA-seq (*n* = 3)	420,000 cells/cm^2^	*miR-99a-5p*, *miR-130a*, *miR-183-5p*, *miR-182-5p*, *miR-663a*	*miR-139-5p*, *miR-145-5p*, *miR-100-5p*, *miR-125b-5p*, *miR-200c-3p*, *miR-3960*	ERK/MAPK, Rho GTPases, PDCD4, FOXO1, BTRC, MMP9, NF-κB, PTEN, SMAD4, TCF4, WNT2B, WNT5A and ZEB1	Invasion, Proliferation and epithelial polarity	[130]
	Luminal A	MCF-7	Microarrays (*n* = 3)	22,000 cells/cm^2^	*miR-149*, *miR-210*, *miR-762*, *miR-548q*, *miR-141*, *miR-1469*, *miR-331-3p*, *miR-1260*, *miR-200a*, *miR-1915*, *miR-429*, *miR-365*, *miR-1908*, *miR-663*, *miR-342-3p*, *miR-150*, *miR-1975*, *miR-132*, *miR-425*, *miR-222*, *miR-193a-3p*, *miR-193b*, *miR-1280*, *miR-22*	*miR-605*, *miR-let-7c*, *miR-7*, *miR-1308*, *miR-let-7g*, *miR-let-7b*, *miR-let-7e*, *miR-181b*, *miR-let-7p*, *miR-let-7a*, *miR-1246*, *miR-let-7i*, *miR-1977*, *miR-629*, *miR-1228*, *miR-203*, *miR-let-7d*, *miR-877*, *miR-342-5p*, *miR-1978*, *miR-1275*, *miR-27b*, *miR-940*, *miR-21*	---	Morphogenesis and invasiveness	[138]
			miRNA-seq (*n* = 3)	400,000 cells/cm^2^	*miR-30b*, *miR-1260b*, *miR-210*, *miR-1246*, *miR-1260*, *miR-34a*, *miR-720*, *miR-1274b*, *miR-4286*, *miR-1290*	*miR-29b-3p*, *miR-17*, *miR-29a*, *miR-1228*, *miR-197*, *miR-940*, *miR-572*, *miR-1234*, *miR-H1*, *miR-766*, *miR-494*, *miR-1973*	AKT2, PI3K, BCL-2, DNMT3B and MYC	Radioresistance and angiogenesis	[109]
			RT-qPCR (*n* = 3)	3000 cells/cm^2^	---	*miR-29c*	DNMT3B, FOXO1, TIMP3, STAT1 and MYC	Proliferation, Invasion and Migration	[119]
	TNBC	MDA-MB-231	Microarrays (*n* = 3)	22,000 cells/cm^2^	*miR-1246*, *miR-654-5p*, *miR-663*, *miR-1469*, *miR-1915*, *miR-762*, *miR-149*, *miR-1826*, *miR-1908*, *miR-575*, *miR-1231*, *miR-1975*, *miR-1977*, *miR-1978*, *miR-638*, *miR-1275*, *miR-150*, *miR-1974*, *miR-128-1*, *miR-193a-5p*, *miR-let-7g*, *miR-27b*	*miR-1308*, *miR-301a*, *miR-381*, *miR-140-3p*, *miR-1280*, *miR-18a*	----	Morphogenesis and Invasion	[139]
			RT-qPCR (*n* = 3)	3000 cells/cm^2^	---	*miR-29c*	DNMT3B, FOXO1, TIMP3 and STAT1	Proliferation, Invasion and Migration	[124]
			RT-qPCR (*n* = 3)	400,000 cells/mL	---	*miR-5683*	ACLY, RACGAP1, AK4, MRPL51, CYB5B, MKRN1, TMEM230, NUP54, ANAPC13, PGAM1 and SOD1	Metabolism	[140]
		MDA-MB-436	RT-qPCR (*n* = 3)	3000 cells/cm^2^	---	*miR-29c*	DNMT3B, FOXO1, TIMP3, STAT1	Proliferation, invasion, migration	[119]
		BT-549	RT-qPCR (*n* = 3)	400,000 cells/mL	---	*miR-5683*	ACLY, RACGAP1, AK4, MRPL51, CYB5B, MKRN1, TMEM230, NUP54, ANAPC13, PGAM1 and SOD1	Metabolism	[140]
**Geltrex^®^**	HER-2+	SK-BR-3	Microarrays (*n* = 3)	21,000 cells/cm^2^	*miR-6529-5p*, *miR-122-5p*, *miR-410-3p*, *miR-409-3p*, *miR-369-3p*	*miR-449c-3p*, *miR-449b-3p*, *miR-3689a-3p*, *miR-449a*, *miR-1247-5p*	WNT, MAPK and AGE-RAGE	Morphogenesis Proliferation and Signaling	[2]
		BT-474	Microarrays (*n* = 3)	15,000 cells/cm^2^	*PIP4K2B*, *FAM3B*, *TMSB4XP1*, *CST1*, *TMSB4X*, *RAD51C*, *AFF3*, *UPK1A*, *ACSF2*, *TMEM150C*	*TMSB4XP6*, *PCGF2*, *ZNF254*, *MDK*, *PRPS2*, *CST4*, *LRRN1*, *TFF3*, *VN1R53P*, *NPY1R*	Diverse gene regulation	Expression Regulation and proliferation	[23]
**Cultrex^®^**	TNBC	4T1 (murine)	RT-qPCR (*n* = 3)	500,000 cells/mL	*miR-181a*	—	TGF-β, SRC, AKT and ERK 1/2	Tumor invasion and matrix remodeling	[92]
		MDA-MB-231							
**PCL**	TNBC	MDA-MB-231	RNA-seq (*n* = 3)	Not specified	*miR-210*, *miR-27a*, *miR-146a*, *miR-619-5p*, *miR-1260*, *miR-1237f*, *miR-1538*	*miR-1908*, *miR-7974*, *miR-671-5p*, *miR-769-3p*, *miR-4750-5p*, *miR-5587-3p*, *miR-0386-3p*, *miR-0415-3p*, *miR-1268b*, *miR-877-3p*, *miR-139-3p*, *miR-4758-3p*, *miR-1915-5p*, *miR-3940-5p*	HIF1A, NF-κB and EMT genes	Stemness and metastasis	[10]

**Table 7:** miRNAs shared between 3D culture methods or molecular subtypes of breast cancer.

miRNA	Type Regulation	Culture Method	Cell Line	Molecular Subtype	Platform	Reference
***miR-22***	Up	Spheroids	MCF-7	Luminal A	RT-qPCR	[134]
					Microarrays	[59]
			MDA-MB-231	TNBC	RT-qPCR	[133]
		Mammospheres	PDTO	Luminal A	Microarrays	[67]
		Matrigel^®^	MCF-7	Luminal A	Microarrays	[139]
	Down		HMT-3522 S1	Non-tumor/ Progression	RNA-seq	[132]
***miR-210***	Up	Matrigel^®^	MCF-10A.B2	Non-tumor/ Progression	Microarrays	[72]
			MCF-7	Luminal A	Microarrays	[139]
					miRNA-seq	[124]
		PCL	MDA-MB-231	TNBC	RNA-seq	[10]
***miR-301a***	Down	Spheroids	MCF-7	Luminal A	RNA-seq	[64]
						[134]
			HCC1395	TNBC	RNA-seq	[64]
		Matrigel^®^	HMT-3522 S1	Non-tumor/ Progression	RNA-seq	[132]
					Microarrays	[141]
			MDA-MB-231	TNBC	Microarrays	[139]
***miR-369***	Up	Spheroids	MCF-7	Luminal A	RNA-seq	[64]
						[134]
			HCC1395	TNBC	RNA-seq	[64]
		Matrigel^®^	HMT-3522 S1	Non-tumor/ Progression	Microarrays	[141]
		Geltrex^®^	SK-BR-3	HER-2+	Microarrays	[2]
***miR-143***	Up	Spheroids	MCF-7	Luminal A	RNA-seq	[134]
	Down	Mammospheres	PDTO	Luminal A	Microarrays	[67]
				Luminal B		
		Matrigel^®^	HMT-3522 S1	Non-tumor/ Progression	RNA-seq	[132]
***miR-145***	Up	Spheroids	MCF-7	Luminal A	RNA-seq	[134]
	Down	Mammospheres	PDTO	Luminal A	Microarrays	[67]
				Luminal B		
		Matrigel^®^	HMT-3522 S1	Non-tumor/ Progression	miRNA-seq	[130]
***miR-181a***	Down	Spheroids	MCF-7	Luminal A	Microarrays	[59]
					RNA-seq	[134]
		Mammospheres	PDTO	Luminal A	Microarrays	[67]
				Luminal B		
		Matrigel^®^	MCF-10A.B2	Non-tumor/ Progression	Microarrays	[72]
		Cultrex^®^	MDA-MB-231	TNBC	RT-qPCR	[82]
***miR-221***	Down	Spheroids	MCF-7	Luminal A	Microarrays	[59]
					RNA-seq	[134]
		Matrigel^®^	MCF-10A.B2	Non-tumor/ Progression	Microarrays	[72]
***miR-27b***	Down	Spheroids	MCF-7	Luminal A	RNA-seq	[135]
		Matrigel^®^	MCF-10A.B2	Non-tumor/ Progression	Microarrays	[72]
			MCF-7	Luminal A		[139]
	Up		MDA-MB-231	TNBC		
***miR-1246***	Up	Spheroids	MCF-7	Luminal A	RNA-seq	[134]
					miRNA-seq	[124]
		Matrigel^®^	MDA-MB-231	TNBC	Microarrays	[139]
	Down		MCF-7	Luminal A		
***miR-99a***	Up	Spheroids	MCF-7	Luminal A	RNA-seq	[14]
		Matrigel^®^	MCF-10A.B2	Non-tumor/ Progression	Microarrays	[72]
			HMT-3522 S1		RNA-seq	[132]
					miRNA-seq	[130]
***miR-100***	Down	Spheroids	MCF-7	Luminal A	RNA-seq	[134]
		Matrigel^®^	HMT-3522 S1	Non-tumor/ Progression	miRNA-seq	[130]
	Up		MCF-10A.B2	Non-tumor/ Progression	Microarrays	[72]
***miR-663***	Up	Mammospheres	PDTO	Luminal A	Microarrays	[67]
				Luminal B		
		Matrigel^®^	HMT-3522 S1	Non-tumor/ Progression	miRNA-seq	[130]
			MCF-7	Luminal A	Microarrays	[139]
			MDA-MB-231	TNBC		

**Table 8:** lncRNAs expressed in breast cancer cells in the different 3D culture methods.

Culture Method	Molecular Subtype	Cell Line	Platform	No. Cultured Cells	Increased lncRNAs	Decreased lncRNAs	Target Genes/ Pathways	Functional Effects	Reference
**Spheroids**	Luminal A	MCF-7	Microarrays (*n* = 3)	80,000 cells/cm^2^	*LINC01790*, *LINC00362*, *LINC01213*, *LINC00996*, *LINC02380*, *LINC01983*, *LINC01310*, *LINC00299*, *LINC01794*, *LINC01258*	*LINC00052*, *LINC01235*, *PURPL*, *LINC02233*, *LINC00869*, *LINC00623*, *LINC01239*, *LINC00492*, *LINC02067*, *LINC02367*	TGFB3, SOX9, FZD4, WNT4, SOX12, TGFB2, PPP2R2B, CCNDBP1, TGFB1, MVD, HMGCS2, ACAT, TANK and PARP9	Mevalonate pathway and Cholesterol and stearate synthesis	[17]
			RNA-seq (*n* = 3)	80,000 cells/cm^2^	*LINC00467*, *MCM3AP-AS1*, *PVT1*, *H19*, *MALAT1*, *GAS5-AS1*, *TP53TG1*, *MEG3*, *TUG1*, *ZFAS1*, *CAPN7-1:2*, *CPEB4-6:1*, *OSBPL6-2:6*, *IGSF5-1:1*, *MLN-1:2*, *RIMKLB-1:1*, *LGALS14-1:2*, *TRIM43-1:1*, *SCYL1-1:4*, *TCL1B-1:1*	*FCGR1B-1:1*, *AC006372.1-2:1*, *CAMK1G-1:2*, *TMEM51-AS2*, *RP11-1070N10.3.1*, *PLGLB2-1:7*, *DUOXA1-1:1*, *DBN1-2:4*, *WRNP1-2:16*, *LMNTD2*	MYC, P53, mTOR and HIF1A	Ubiquitination, cell survival and adhesion	[143]
**Matrigel^®^**	Tumor progression model	MCF-10A-HRAS subclones	RNA-seq (*n* = 2)	8000 cells/mL	*LINC00973*, *AC019172.2*, *LINC02154*, *LINC00431*, *RP11-24N18.1*, *RP11-217E22.5*, *RP11-879F14.2*, *RP11-3L8.3*, *RP11-626H12.2*, *LINC01705*	*AC009299.3*, *RP11-463O9.5*, *RP3-460G2.2*, *RP1-124C6.1*, *RP11-74E22.3*, *LINC00640*, *RP11-326C3.7*, *RP11-1000B6.5*, *AC098617.1*, *RP4-737E23.2*	PDCD4, FOXC2, SLC2A9, CCNDBP1 and ICAM	Tumor progression, Invasion and metastasis	[129]
	Luminal A	MCF-7/LCC2 MCF-7/LCC9	RT-qPCR (*n* = 3)	600,000 cells/mL	*H19*	---	NOTCH and C-MET	Resistance to endocrine therapy	[96]
	TNBC	MDA-MB-231	RT-qPCR (*n* = 3)	22,000 cells/cm^2^	*HOTAIR-N*	---	integrin α2 and SRC	cell growth and invasion	[144]
		HS-578T	RT-qPCR (*n* = 3)	22,000 cells/cm^2^	*HOTAIR-N*	---	integrin α2 and SRC	cell growth and invasion	[144]
			RT-qPCR (*n* = 3)	10,000 cells/well	*AFAP1-AS1*	---	---	Hypoxia	[97]
	PDX	MMTV-PyMT, MMTV-Neu-NDL	RNA-seq (*n* = 3)	Not specified	*Rik-203*, *Gm16025*, *A330074K22Rik*, *Gm13387*, *RP23-437C24.2*, *RP23-327I19.1*	---	Androgen receptor and P63	Proliferation and Invasiveness	[77]
**Geltrex^®^**	Luminal B	BT-474	Microarrays (*n* = 3)	15,500 cells/cm^2^	*RP11-20F24.2*, *UCA1*, *LASP1NB*, *CTD-2566J3.1*, *LINC00847*	*XIST*, *CYTOR*, *LINC00857*, *MIR4435-2HG*	EGFR, TGFβ, FOXA1 and GINS2	Proliferation, migration, invasion, metastasis, drug resistance, glycolysis and OXPHOS	[23]

## Abbreviations

The following abbreviations are used in this manuscript:

ER+	Estrogen Receptor positive
PR+	Progesterone Receptor positive
HER-2+	Human Epidermal Growth Factor Receptor 2 HER2
TNBC	Triple-negative
2D	Two-dimensional
EMT	Epithelial–mesenchymal transition
3D	Three-dimensional
miRNAs	MicroRNAs
lncRNAs	Long non-coding RNAs
circRNAs	Circular RNAs
ROS	Oxidative stress
KRT15	Keratin 15
FABP5	Fatty acid-binding protein 5
AGR2	Anterior gradient protein 2 homolog
ERBB2	Erythroblastic Oncogene B2
PPARG	Peroxisome Proliferator-activated Receptor Gamma
FADS1	Fatty Acid Desaturase 1
PTGER4	Prostaglandin E Receptor 4
PARG	Poly(ADP-ribose) Glycohydrolase
PALM2	Paralemmin
ULA	Ultra-low adhesion
PolyHEMA	Poly-2 hydroxyethyl methacrylate
CSC	Cancer stem cell
ECM	Extracellular matrix
PDTOs	Patient-derived tumor organoids
PDX	Xenograft-Derived Organoids
OoC	Organ-on-a-Chip
EHS	Engelbreth-Holm-Swarm
lrECM	High laminin content and form the extracellular matrix
DDR2	Receptor 2 containing the discoidin domain
STAT1	Signal transducer and activator of transcription 1
P27	Cyclin-dependent kinase inhibitor 1B
GFRM	Reduced growth factor content
ERK1/2	Extracellular signal-regulated kinase 1/2
PHB	Polyhydroxybutyrate
dECM	Decellularized extracellular matrix
PEG	Polyethylene glycol
PLG	Poly(lactide-co-glycolide)
PCL	Poly(ε-caprolactone)
PAM	Polyacrylamide
GelMA	Methacryloyl gelatin
GelSH	Thiolated gelatin crosslinked with PEG-4MAL
PLGA	Poly(lactic-co-glycolic acid)
PDMS	Polydimethylsiloxane
FA	Focal adhesions
WNT	Wingless-related integration site
ILK	Integrin-linked kinase
MYPT1	Myosin phosphatase subunit 1
MST1/2	Mammalian sterile 20-like kinase 1/2
LATS1	Large tumor suppressor kinase 1
FAK	Focal adhesion kinase 1
SRC	Pro-oncogenic tyrosine-protein kinase SRC
TRPV4	Transient receptor potential cation channel subfamily V member 4
YAP	Yes-associated protein
DNMT3B	DNA (cytosine-5)-methyltransferase 3B
HOX	Homeobox
BRD4	Bromodomain-containing protein 4
RBL1	Retinoblastoma-like protein 1
MAPK	Mitogen-Activated Protein Kinase
NOTCH	Neurogenic locus notch homolog
SBDS	SBDS ribosome maturation factor
TMPRSS2	Transmembrane serine protease 2
PAX5	Paired box 5
CREBBP	Histone acetyltransferase CREB-binding protein
PI3K	Phosphatidylinositol 3-kinase
PTEN	Phosphatase and tensin homolog
HDAC	Histone deacetylases
HAT	Histone acetyltransferases
TADs	Tumor-associated domains
DDX21	DExD-box helicase 21
CTCF	CCCTC-binding factor
TEAD	Transcriptional Enhanced Associate Domain
FOXA1	Forkhead box A1
EZH2	Enhancer of Zeste Homolog 2
HMGB1	High Mobility Group Box 1
RBPs	RNA-binding proteins
Cx43	Connexin 43
FOXO1	Forkhead box protein O1
TP73	Tumor protein p73
TP63	Tumor protein p63
TP53	Tumor protein p53
AGO2	Argonaute RISC catalytic component 2
BRAF	B-Raf proto-oncogene, serine/threonine kinase
HIF1A	Hypoxia-inducible factor 1-alpha
MYC	MYC proto-oncogene
E2F	E2F transcription factor
FOXO3	Forkhead box O 3
BVES	Blood vessel epicardial substance
ARHGAP17	Rho GTPase activating protein 17
OXPHOS	Oxidative phosphorylation
PDCD4	Promoting programmed cell death protein 4
ZEB1	Zinc Finger E-Box Binding Homeobox 1
BCL2	BCL2 apoptosis regulator
GLI3	GLI family zinc finger 3
KLF4	Kruppel-like factor 4
SMAD2	Mothers against decapentaplegic homolog 2
SOX4	SRY-box transcription factor 4
SP1	Sp1 transcription factor or Specificity Protein 1
BMP2	Bone Morphogenetic Protein 2
TGFBR1	Transforming growth factor beta receptor 1
KRAS	Kirsten rat sarcoma viral oncogene homolog
ERBB4	Receptor tyrosine-protein kinase erbB-4
VEGFA	Vascular Endothelial Growth Factor A
KLF5	Kruppel-like factor 5
RASA1	RAS p21 protein activator 1
CDH2	Cadherin-2
FGF1	Fibroblast growth factor 1
MAP3K7	Mitogen-activated protein kinase kinase kinase 7
VEZF1	Vascular endothelial zinc finger 1
ZFP36L1	ZFP36 ring finger protein like 1
BTRC	Ubiquitin protein ligase E3 containing beta-transducin repeats
TGF-β2	Transforming growth factor beta 2
AKT2	AKT serine/threonine kinase 2
TIMP3	Tissue inhibitor of metalloproteinase 3
ACLY	ATP citrate lyase
RACGAP1	Rac GTPase-activating protein 1
AK4	Adenylate kinase 4
MRPL51	Mitochondrial ribosomal protein L51
CYB5B	Cytochrome b5 type B
MKRN1	Makorin ring finger protein 1
TMEM230	Transmembrane protein 230
NUP54	Nucleoporin 54
ANAPC13	Anaphase-promoting complex subunit 13
PGAM1	Phosphoglycerate Mutase 1
SOD1	Superoxide dismutase 1
AGE	Advanced glycation end-product
RAGE	Receptor for advanced glycation end-product
NF-κB	Nuclear Factor kappa-light-chain-enhancer of activated B cells
TGF-β3	Transforming Growth Factor Beta 3
SOX9	SRY-Box Transcription Factor 9
FZD4	Frizzled Class Receptor 4
WNT4	Wnt Family Member 4
SOX12	SRY-Box Transcription Factor 12
PPP2R2B	Protein Phosphatase 2 Regulatory Subunit Bβ
CCNDBP1	Cyclin-D1 Binding Protein 1
MVD	Mevalonate Diphosphate Decarboxylase
HMGCS2	3-Hydroxy-3-Methylglutaryl-CoA Synthase 2
ACAT	Acetyl-CoA Acetyltransferase
TANK	TRAF Family Member-Associated NF-κB Activator
PARP9	Poly(ADP-ribose) Polymerase Family Member 9
FOXC2	Forkhead Box C2
SLC2A9	Solute Carrier Family 2 Member 9
CCNDBP1	Cyclin D1 Binding Protein 1
ICAM	Intercellular Adhesion Molecule
C-MET	MET Proto-Oncogene, Receptor Tyrosine Kinase
EGFR	Epidermal Growth Factor Receptor
